# Different effectiveness of acupuncture treatment schedule on ART pregnancy outcomes: a systematic review and network meta-analysis

**DOI:** 10.3389/fendo.2025.1602710

**Published:** 2025-09-05

**Authors:** Yunhong Yang, Huan Chen, Han Tang, Hongjun Kuang, Yi Gou, Hong Zhao

**Affiliations:** ^1^ Department of Acupuncture and Moxibustion, Shenzhen Hospital of Shanghai University of Traditional Chinese Medicine, Shenzhen, China; ^2^ Institute of Acupuncture and Moxibustion, China Academy of Chinese Medical Sciences, Beijing, China

**Keywords:** acupuncture, assisted reproductive technology, pregnancy outcome, systematic review, meta-analysis

## Abstract

**Background:**

Acupuncture shows potential in enhancing pregnancy outcomes in Assisted Reproductive Technology (ART), yet the variability in protocols necessitates identifying optimal strategies.

**Objective:**

This study aimed to evaluate the effects of different acupuncture treatment schedules—specifically the timing, duration, and frequency—on ART pregnancy outcomes, and to identify the most effective strategies.

**Methods:**

A systematic review and network meta-analysis of randomized controlled trials published up to May 2024 was conducted. Studies assessing acupuncture’s impact on ART outcomes were included. Primary outcomes were clinical pregnancy rate and live birth rate; secondary outcomes included fertilization rate and high-quality embryo rate.

**Results:**

Acupuncture significantly improved clinical pregnancy rate (RR 1.26), live birth rate (RR 1.10), fertilization rate (RR 6.64), and high-quality embryo rate (RR 12.67). Ranking analysis indicated the embryo culture period as the most effective treatment timing, followed by the ovarian stimulation and ART preparation periods. Longer treatment durations (≥3 months) and higher session numbers (≥20) yielded superior outcomes.

**Conclusions:**

These findings underscore the importance of personalized acupuncture protocols to optimize ART success. Tailoring acupuncture protocols based on timing, duration, and frequency may optimize reproductive outcomes in women undergoing ART.

## 1 Introduction

Assisted Reproductive Technology (ART) encompasses a variety of medical techniques designed to assist individuals facing reproductive challenges. Widely utilized in infertility treatment, ART includes techniques such as *in vitro* fertilization (IVF), intracytoplasmic sperm injection (ICSI), and the preservation of embryos or eggs through freezing (1, 2). ART is an important treatment for infertile couples{sp} (3–5){/sp}. More than 8 million individuals have been conceived through ART techniques worldwide, and the proportion of ART newborns to the total number of newborns continues to increase in different regions or countries (6, 7).

Globally, the clinical pregnancy rate (CPR) ranges from 29% to 35% (8), with a live birth rate (LBR) of approximately 30% (9). In China, the CPR is reported at 51.95% and the LBR at 42.39% (10). Despite these advancements, 10-15% of patients experience repeated implantation failure after multiple embryo-transfers (ETs) (11, 12). The economic burden imposed by repeated treatment cycles and high-cost interventions imposes substantial psychological stress on patients (13). Optimization of CPR and enhancement pregnancy outcomes to reduce both cycles frequency and financial strain have consequently emerged as priority objectives in ART (14). Studies highlight patient age and ART techniques as key factors influencing CPR (15). Notably, a real-world cohort study by Yang Huisheng demonstrated that acupuncture-assisted ART improved pregnancy rates in patients with premature ovarian insufficiency (16).

Complementary and alternative medicine, including acupuncture, is increasingly employed to improve CPR and LBR in ART patients (17). In the United States, 47% of individuals undergoing infertility patients receive acupuncture during in IVF-ET cycles (18). A retrospective study from Harran University in Turkey found acupuncture increased CPR from 33.3% to 60.9% and LBR from 30.0% to 71.7% in IVF-ET patients with unexplained infertility (19). Similarly, a prospective trial reported markedly higher pregnancy rate (64.7%) in women receiving acupuncture alongside ART compared to 42.5% in those who did not (20). In China, acupuncture improved both LBR (54.5% vs. 41.0%) and CPR (58.2% vs. 52.5%) in patients with recurrent implantation failure (21). Over the past five years, five systematic reviews have evaluated acupuncture efficacy in ART{sp} (22–25){/sp}. However, due to low-quality evidence, varying protocols, and multiple influencing factors, no definitive conclusions can be drawn, and acupuncture is not yet recommended in evidence-based guidelines (26).

Acupuncture is commonly utilized as an adjunctive therapy in ART, but standardized intervention protocols remain undefined. In the U.S., acupuncture is typically administered before and on the day of embryo transfer, while in China, it often begins before the ART cycle starts. There is also considerable variation in treatment frequency and duration. In the U.S. and Europe, acupuncture may be given 2 to 3 times per week, while in China, it is usually 5 to 6 times per week (27). Systematic reviews have reported treatment durations ranging from 1 day to 4 months, with the number of sessions varying from a minimum of 1 to over 35. Some studies suggest that the dosage of acupuncture is a critical factor influencing its effectiveness (28).

Therefore, we intend to use network meta-analysis to clarify the optimal stimulation parameters for acupuncture intervention in ART, including the timing of intervention, the total number of acupuncture sessions, the total duration of acupuncture, and the treatment course, to provide evidence-based support for optimizing acupuncture protocols and improving therapeutic efficacy.

## 2 Materials and methods

### 2.1 Protocol and registration

This study is reported following the PRISMA statement and is registered on PROSPERO (https://www.crd.york.ac.uk/PROSPERO/view/CRD42024547113, registration number: CRD42024547113)).

### 2.2 Literature search strategy

Computer searches were performed on PubMed, Embase, Cochrane Library, Web of Science, China Biomedical Literature Service System (SinoMed), China Knowledge Network (CNKI), Wanfang Data Knowledge Service Platform (Wanfang Data), and Weipu Chinese Scientific and Technical Journal Database (VIP). This study encompassed literature published from database inception through May 2024. The research adopts a combination of subject terms and free words to develop a tailor-made search strategy for each database. The main subject terms/free words involved were *in vitro* fertilization, acupuncture and randomized controlled trials. The language was limited to Chinese and English.

### 2.3 Inclusion criteria

#### 2.3.1 Type of study

Randomized controlled trial (RCT).

#### 2.3.2 Study population

Female patients who were infertile and underwent ART, included ICSI or IVF or ET and planned for fresh or frozen embryo transfer. There were no restrictions on infertility diagnostic criteria or age.

#### 2.3.3 Interventions

The treatment group underwent at least one acupuncture treatment (including electroacupuncture). Acupuncture therapy was used alone and not combined with any other adjuvant therapy (e.g. moxibustion, cupping, Chinese herb). The control group consisted of a blank control (conventional treatment regimen only without any other adjuvant therapies) and placebo acupuncture (using a placebo needle or device, needling on non-meridian-non-acupuncture points). The conventional treatment regimen for ART (including conventional hormone replacement therapy, superovulation long protocol, etc.) was the same for both groups.

#### 2.3.4 Outcomes

The study evaluates key ART outcomes, including Clinical Pregnancy Rate, Live Birth Rate, Fertilization Rate, and High-Quality Embryo Rate. CPR is the percentage of clinical pregnancies confirmed by positive hCG tests 14 days post-embryo-transfer and ultrasound detection of a gestational sac with a fetal heartbeat by day 34. LBR reflects the proportion of live births, defined as deliveries after 24 weeks’ gestation, relative to total births. Fertilization Rate represents the percentage ratio of fertilized oocytes to total retrieved oocytes. High-Quality Embryo Rate is determined by evaluating embryos on day 3 post-fertilization based on morphology, cleavage sphere uniformity, and fragmentation rate. Grades 1 and 2 embryos, characterized by uniform spheres, regular morphology, and fragmentation below 25%, are considered high quality. The rate is calculated as the number of Grade 1 and 2 embryos divided by the total embryos, multiplied by 100%. These metrics provide a comprehensive assessment of ART success (1).

### 2.4 Exclusion criteria

The following criteria will be applied to exclude studies that do not meet the requirements of this review: ① Non-infertile patients or studies failing to provide specify clinical indications for IVF, with unsuccessful author contact attempts. ② Studies that do not provide a detailed treatment plan, and where the authors cannot be reached for further information. Exclusion will occur if the study data and protocol are not made available upon request. ③ Literature that has been published more than once, or works by the same author that potentially contain overlapping data (identical source and intervention with overlapping timeframes). ④ Systematic evaluations, reviews, opinion pieces, editorials, guidelines, and protocol papers. ⑤ Studies focused on acupuncture analgesia in the context of egg retrieval procedures.

### 2.5 Literature screening and data extraction

Two evaluators independently screened the literature, extracted data and cross-checked according to the above criteria. In case of disagreement, a third researcher assisted in the adjudication. The data were extracted into Excel, and the main contents included: ① Basic information of the included studies. ② Baseline characteristics of the study population, and intervention details (detailed extraction of the treatment protocols of both groups, especially the timing of acupuncture interventions, total duration, total number of sessions, and frequency). ③ Key elements of the risk of bias evaluation. ④ Data on outcome indicators and outcome measures of interest.

We collected factors of acupuncture treatment schedule (T, time of starting acupuncture treatment;D, total duration of treatment; N, total number of treatment sessions; F, frequency of treatment in weeks) from each of the trials. For the convenience of further analysis, classify them according to the following rules based on the experts’ experiences and literature evidences. ① T: ART preparation period, ovarian stimulation period, embryo culture period, and transfer day. ② D: short-duration(less than one menstrual cycle),medium duration (greater than one month and less than 2 months), and long duration(greater than or equal to 2 menstrual cycles). ③ N: less than 10 sessions, between 10 and 20 sessions, more than or equal 20 sessions. ④ F: less than or equal 3 times and more than 3 times a week.

### 2.6 Risk of bias assessment of included studies

To ensure the methodological rigor of the included studies, the Cochrane Risk of Bias Assessment Tool (RoB 2) was used to evaluate the risk of bias across randomized controlled trials (RCTs). The assessment focused on five key domains: the randomization process, deviations from intended interventions, missing outcome data, accuracy of outcome measurement, and selective reporting of outcomes. Each study’s overall risk of bias was categorized as ‘high risk,’ ‘some concerns,’ or ‘low risk.’ The risk of bias for the included studies was independently appraised by two review authors using the RoB 2 tool. In case of discrepancies between the reviewers’ assessments, they will engage in a collaborative discussion to reach a consensus. If this fails, a third researcher was consulted to provide an objective perspective and facilitate a final decision.

The results of the risk of bias assessment were integrated into the interpretation of the findings. Studies judged to have a ‘high risk’ of bias or raising ‘some concerns’ were explicitly noted in the discussion, as their results may be less reliable and could potentially distort the overall conclusions. The potential of bias introduced by methodological limitations in these studies were considered when drawing inferences about intervention effects. Furthermore, sensitivity analyses were performed where appropriate, to assess the robustness of the primary findings.

### 2.7 Data synthesis and analysis

Data will be synthesized and analyzed using Stata 17.0 and Revman V.5.4. Continuous variables will be expressed as mean difference (MD) and standard deviation (SD), while binary variables will be presented as risk ratio (RR) with 95% confidence intervals (CI). Relationships between different acupuncture intervention schemes will be illustrated using network evidence graphs, where lines connecting nodes represent direct comparisons, with line thickness proportional to the number of studies and node size proportional to sample size. The consistency of each closed loop will be evaluated by calculating the inconsistency factor and its 95% CI (29). Inconsistency models will test for discrepancies, and when P > 0.05, a consistency model will be analyzed (30). Intervention effects of different schemes will be ranked using a surface under the cumulative ranking probability plot (SUCRA). Funnel plots will assess publication bias or small sample effects. To identify the optimal timing, duration, number of sessions, and frequency of acupuncture in ART, meta-analysis will synthesize similar study designs, interventions, and outcome indicators. A fixed-effects model will be used for studies with high homogeneity (I² ≤ 50%), while a random-effects model will be used for those with high heterogeneity (I² > 50%). Narrative analyses will be performed for data that cannot be statistically synthesized, and sensitivity analyses will explore possible sources of heterogeneity.

## 3 Results

### 3.1 Results of literature search

**Figure 1:**
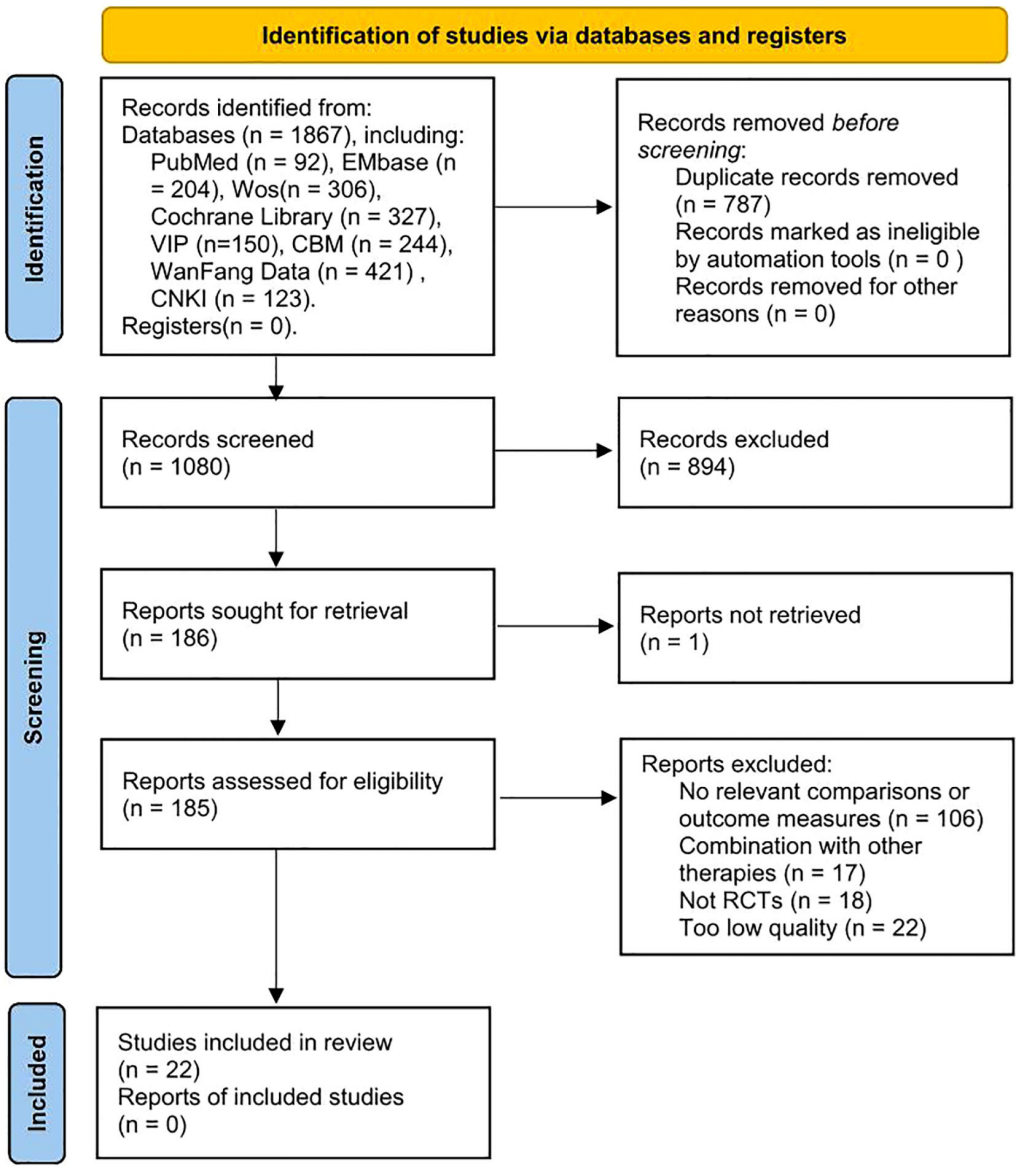
PRISMA flow chart.

An initial review of 1867 relevant literature was conducted, including PubMed (n=92), EMbase (n=204), Cochrane Library (n=327), WOS(n=306), VIP (n=150), CBM (n=244), WanFang Data (n=421) and CNKI (n=123). After a layer-by-layer screening process, 22 RCTs were finally included{sp} (31–52){/sp}.The PRISMA flowchart shows the detailed process of literature selection process (**Figure 1**).

### 3.2 Basic characteristics of the included literature

A total of 22 RCTs involving 3,677 patients from seven countries were included, with 13 studies in English and nine in Chinese. All patients, diagnosed with infertility, underwent ART. Treatment groups received acupuncture or electroacupuncture, while control groups had no adjuvant therapy or sham acupuncture. Acupuncture alone was used in 15 studies (68.18%), and electroacupuncture in 7 (31.82%). Most controls were blank (68.18%). The total duration of acupuncture treatments ranged from a minimum of 1 day to a maximum of 4 months. The total number of acupuncture treatment session ranged from a minimum of 1 session to a maximum of more than 35 sessions. The frequency of acupuncture in weeks was a minimum of 1 session per week and a maximum of 7 sessions per week. The basic characteristics of the included studies are shown in **Supplementary Table 1**.

### 3.3 Results of risk of bias assessment of included studies

The risk of bias evaluation for the included RCTs is detailed in **Supplementary Figures 1**, **2**. Seven studies (31.8%) were rated as “low risk” (32, 33, 36, 37, 39, 47, 48), while 15 (68.2%) had “some concerns”{sp} (34, 35, 38, 40–46, 49–53){/sp}. Allocation concealment, achieved through sealed envelopes or computer-generated sequences, was used in 14 studies (63.6%). Blinding of acupuncture therapists was unfeasible due to the intervention’s nature. Most studies (95.5%) reported complete data and all intended outcomes. Sham acupuncture was used in five studies, lightly stimulating the skin without achieving “qi gain,” while two used needling near but not on true acupuncture points, potentially increasing bias.

### 3.4 Net meta-analysis

#### 3.4.1 Net meta-analysis

There were 22 papers all reporting CPR. Based on the classification of T, the six intervention strategies formed two quadrilateral loops (**Supplementary Figure 3a**). We then used a global inconsistency test to evaluate the overall consistency within the network, which showed a result of P = 0.5057, indicating no significant inconsistency (**Supplementary Table 2**). No closed loops were formed in any of the mesh relationship plots because no comparisons were found between different acupuncture treatment durations or the total number of different acupuncture treatments (**Supplementary Figures 3b, c** **).** A global inconsistency test revealed that there was no significant inconsistency in the net analysis model for different total number of acupuncture sessions (P = 0.0938). There was significant inconsistency in the network analysis model for acupuncture sessions (P = 0.0107) (**Supplementary Table 2**). However, we proceeded to assess the local inconsistency in the network Meta-analysis using the analysis of the node splitting method and found that the tau value was greater than 0.05. This demonstrated that their inconsistency was not significant (**Supplementary Table 3**). The Forest of Consistency map can be seen in **Supplementary Figures 4**–**6**. Therefore, the reliability of the results of this network analysis is high.

#### 3.4.2 Net meta-comparison results

A random-effects model utilizing a frequency-based framework was employed for the analyses. In terms of the impact of T on CPR, the efficacy was ranked as follows: during embryo culture period > ovarian stimulation period > ART preparation period > sham acupuncture > embryo transfer day > no adjuvant treatment (**Supplementary Table 4**). ② Concerning the D on CPR, the efficacy was ranked as follows: long-duration > medium-duration > short-duration > non acupuncture treatment (**Supplementary Table 5**). ③ In terms of the N, the efficacy increased with the frequency of treatments: more than or equal 20 sessions > 10 – 20 sessions > less than 10 sessions > 0 sessions at real acupuncture points(non acupuncture treatment) (**Supplementary Table 6**). We did not perform a net meta-analysis of frequency because not all of the included literature documented the frequency of acupuncture treatments.

#### 3.4.3 Cumulative ranking probability results

The SUCRA results were utilized to determine the optimal probability ranking for each intervention. The clinical pregnancy rate rankings are as follows: ① In terms of T, the embryo culture period was found to be the most effective, outperforming the ovarian stimulation period and the ART preparation period. ② When considering the D, a clear hierarchy emerged: long-duration treatments were superior to medium-duration treatments, which in turn were more effective than short-duration treatments. ③ Regarding the N, the rankings indicated that more than or equal 20 sessions were the most beneficial, followed by 10 – 20 sessions, and then less than 10 sessions (**Supplementary Figures 7a–c**).

#### 3.4.4 Publication bias or small sample effect test.

The publication bias test for the outcome indicators through the funnel plot found that the symmetry of the funnel plot for each outcome indicator was good. The publication bias or small sample effect had a small impact (**Supplementary Figures 8**–**10**).

### 3.5 Effect of acupuncture on CPR

#### 3.5.1 Effect of different time of starting acupuncture treatment on CPR

**Figure 2:**
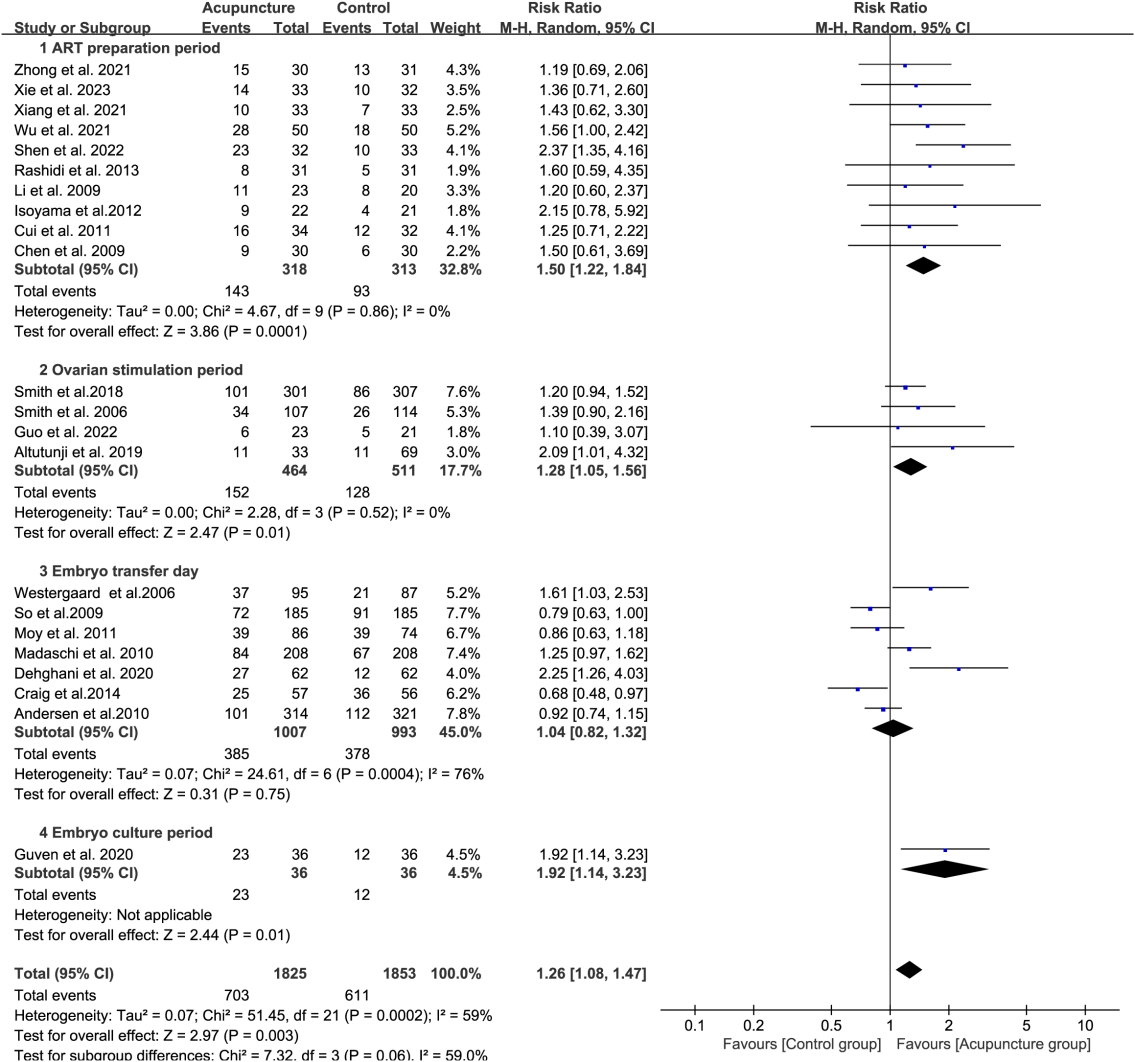
Forest plot of CPR for acupuncture group versus control groups (Differentiation according to the time of the acupuncture intervention).

The CPR was significantly higher in the acupuncture group compared to the control group (P = 0.003, RR 1.26, 95% CI[1.08, 1.47]). Ten studies on acupuncture during the ART preparation period showed the most pronounced effect (RR 1.50, 95% CI[1.22, 1.84], I² = 0%){sp} (32–39, 44, 52){/sp}. Four studies during the ovarian stimulation period also demonstrated improvement (RR 1.28, 95% CI[1.05, 1.56], I² = 0%) (41, 43, 48, 53). In contrast, seven studies focusing on acupuncture on transfer day showed mixed results with high heterogeneity (RR 1.04, 95% CI[0.82, 1.32], I² = 76%){sp} (40, 42, 46, 47, 49–51){/sp}. Notably, three studies (Craig et al., Moy et al., and So et al) (42, 49, 51) demonstrated reduced pregnancy rates with RR values of 0.68 (95% CI[0.48, 0.97]), 0.86 (95% CI[0.63, 1.18]), and 0.79 (95% CI[0.63, 1.00]) respectively. Only one study reported on acupuncture during the embryo culture period, showing significant benefit (RR 1.92, 95% CI[1.14, 3.23], P = 0.01) (45). These findings suggest that acupuncture before or during ART cycles improves CPR more effectively than treatment limited to transfer day (**Figure 2**).

#### 3.5.2 Effect of different total duration of treatment on CPR

**Figure 3:**
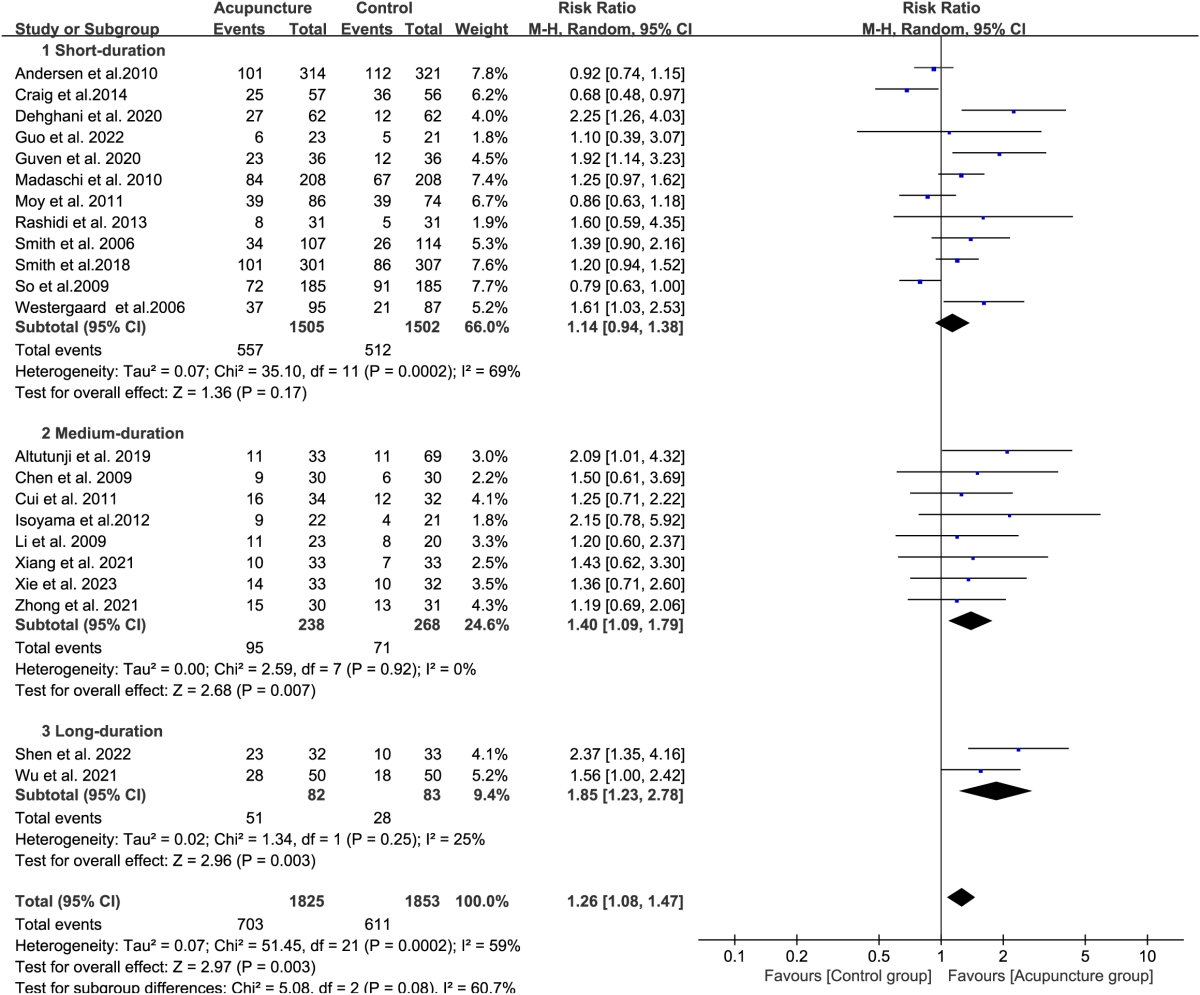
Forest plot of CPR for acupuncture group versus control groups (Differentiation according to the duration of acupuncture).

We analyzed the effect of the D on CPR. Short-duration acupuncture (<1 cycle, 12 studies) showed limited effect (RR 1.14, 95% CI[0.94, 1.38], I² = 69%){sp} (40, 42–51, 53){/sp}. Four studies (Andersen et al., Craig et al., Moy et al., and So et al){sp} (42, 49–51){/sp} under this short-duration regimen reported reduced CPR. Medium-duration (2-3 cycles, 8 studies) showed greater improvement (RR 1.40, 95% CI[1.09, 1.79], I² = 0%){sp} (32, 34–36, 38, 39, 41, 52){/sp}, while long-duration (>3 cycles, 2 studies) also demonstrated significant benefit (RR 1.26, 95% CI[1.08, 1.47], I² = 25%) (33, 37). Longer treatment durations improved ART outcomes more effectively (**Figure 3**).

#### 3.5.3 Effect of different total number of treatment sessions on CPR

**Figure 4:**
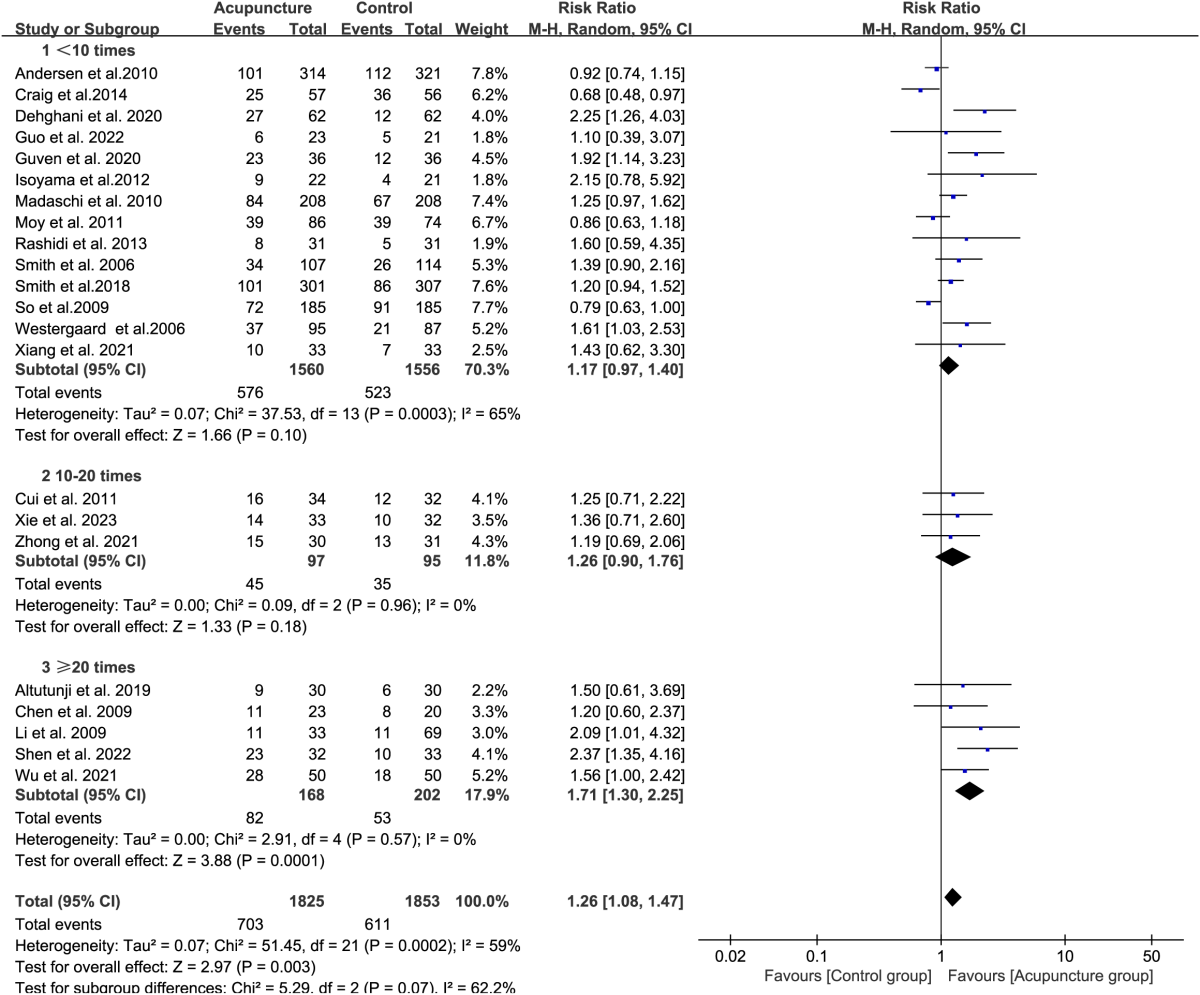
Forest plot of CPR for acupuncture group versus control groups (Differentiation based on the total number of acupuncture sessions).

We analyzed the effect of the N on CPR. There are fourteen studies which the acupuncture treatment are less than 10 sessions (RR 1.17, 95% CI[0.97, 1.40], I²= 65%){sp} (38, 40, 42–53){/sp}. Among these, four studies (Andersen et al., Craig et al., Moy et al., and So et al){sp} (42, 49 –51){/sp} reported reduced CPR following acupuncture treatment. There are 3 studies which participants accepted 10 to 20 acupuncture treatment sessions (RR 1.26, 95% CI[0.90, 1.76], I²= 0%) (32, 36, 39), and five studies are 20 or more treatment sessions (RR 1.71, 95% CI[1.30, 2.25], I²= 0%){sp} (33–35, 37, 41){/sp} (**Figure 4**). These results suggest that a higher total number of acupuncture treatments is associated with more favorable clinical pregnancy outcomes in ART.

#### 3.5.4 Effect of different frequency of treatment in weeks on CPR

**Figure 5:**
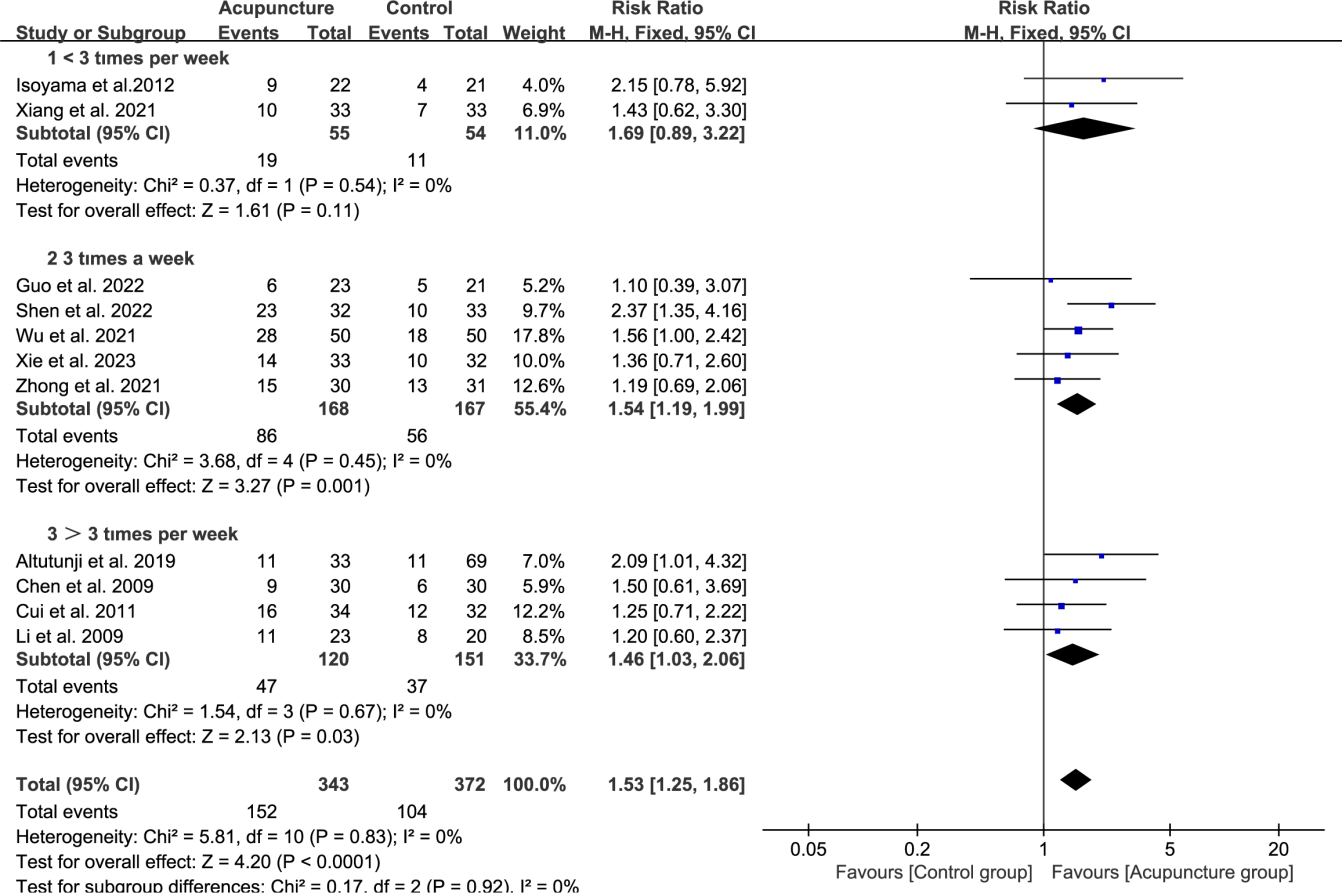
Forest plot of CPR for acupuncture group versus control groups (Differentiation according to the frequency of acupuncture treatment).

The frequency of acupuncture was less than 3 sessions in 2 studies (RR 1.69, 95%CI[0.89, 3.22], I^2^ = 0%) (38, 52). The weekly frequency of acupuncture was approximately equal to 3 sessions in 5 studies (RR 1.54, 95%CI[1.19, 1.99], I^2^ = 0%) (32, 33, 36, 37, 53). The weekly frequency of acupuncture was greater than 3 sessions in 4 studies (RR 1.46, 95%CI[1.03, 2.06], I^2^ = 0%) (34, 35, 39, 41) (**Figure 5**). The results indicated that the clinical pregnancy rate outcome of ART was optimal when the frequency was equal to 3 sessions.

### 3.6 Effect of acupuncture on LBR

**Figure 6:**
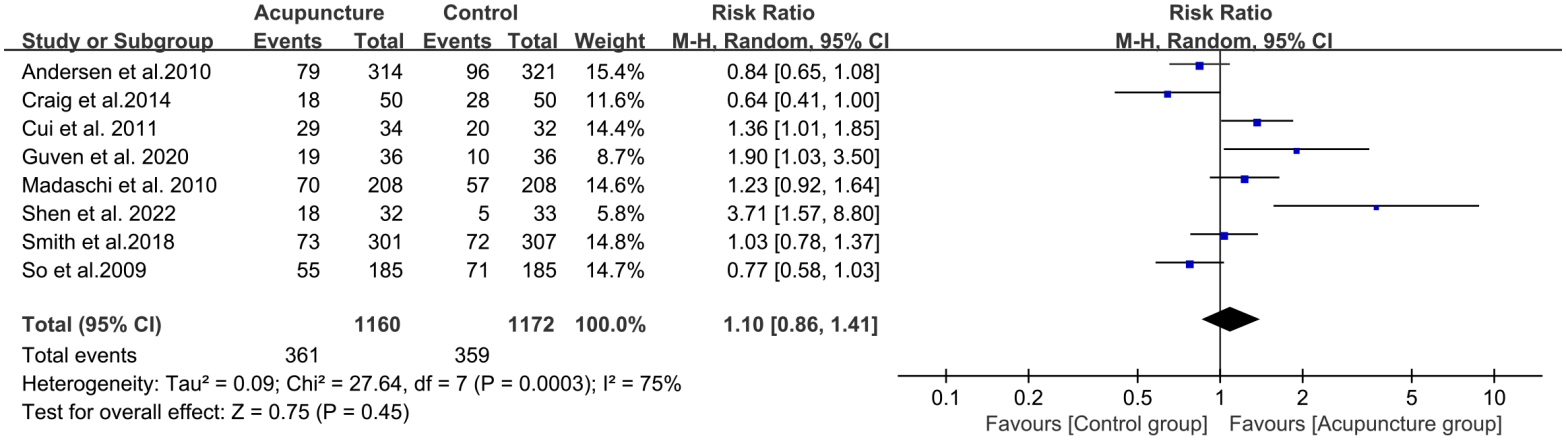
Forest plot of LBR for acupuncture group versus control groups.

There are 8 literature studies on the efficacy of acupuncture on live birth rate, which showed no statistical difference (P > 0.05) compared to the control group. This may be due to the high heterogeneity of these 8 studies (RR 1.10, 95%CI[0.86, 1.41], I^2^ = 75%){sp} (37, 39, 45, 46, 48–51){/sp} (**Figure 6**). Among them, three studies (Andersen et al., Craig et al., and So et al.){sp} (49–51){/sp} suggested that acupuncture did not favor increasing live birth rates, while the remaining five studies indicated that acupuncture was beneficial for improving LBR. The substantial heterogeneity in live birth rate (I²=75%) primarily stems from marked divergences in acupuncture protocols across the eight trials reporting this outcome. Among the included studies, six implemented acupuncture regimens with a total treatment session count <10, while two studies utilized medium-high dose protocols. The most intensive regimen involved daily acupuncture administration.

### 3.7 Effect of acupuncture on fertilization rate

**Figure 7:**
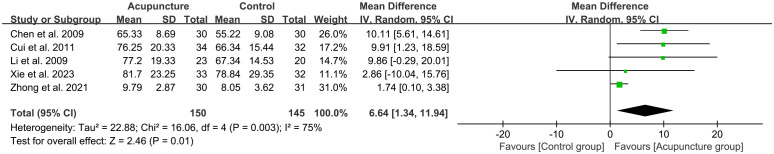
Forest plot of fertilization rate for acupuncture group versus control groups.

A total of five studies{sp} (32, 34–36, 39){/sp} observed the effect of acupuncture on fertilization rate, with a total sample size of 295 in the acupuncture and control groups. The results of Meta-analysis suggested that the fertilization rate in the acupuncture group was significantly higher than that in the control group, and the comparison was statistically different (P = 0.01) (**Figure 7**). However, the heterogeneity of the study was high (RR 6.64, 95%CI[1.34, 11.94], I^2^ = 75%). Heterogeneity in fertilization rate was predominantly attributable to acupuncture frequency divergence, including three sessions per week (n=2), five sessions per week (n=2), and seven sessions per week (n=1).

### 3.8 Effect of acupuncture on the rate of high-quality embryos

**Figure 8:**
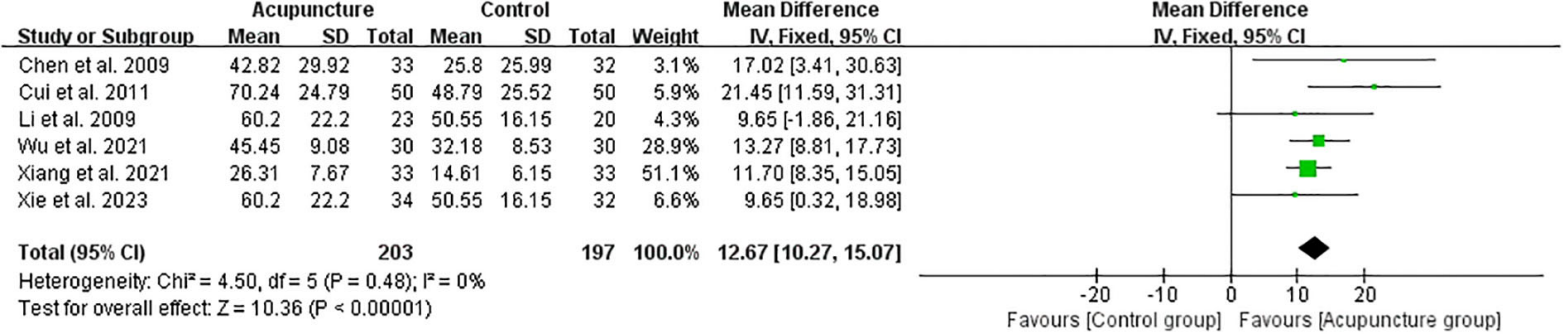
Forest plot of High-quality embryo rate for acupuncture group versus control groups.

A total of six studies{sp} (32–35, 38, 39){/sp} documented the effect of acupuncture on the rate of high-quality embryos. The total sample size of the acupuncture and control groups was 400. The results of the studies suggested that the rate of high-quality embryos in the acupuncture group was significantly higher than that in the control group, with a statistically significant difference (RR 12.67, 95%CI[10.27, 15.07], I^2^ = 0%) (**Figure 8**).

### 3.9 Sensitivity analysis

The “Paulus protocol” is a protocol of needling 25 minutes before and after ET (54). The number of days of acupuncture is only 1. We excluded transfer-day acupuncture studies (n=7) from sensitivity analyses due to fundamentally inadequate treatment duration. The occurrence and development of acupuncture effects require a certain duration, exhibiting specific patterns over time. This relationship between acupuncture efficacy and time is termed the "temporal characteristics of acupuncture efficacy”. The temporal curve of acupuncture efficacy divides the initiation and progression of effects into three phases: latency period, efficacy period and Residual-effect period (55). However, Our analysis identified 7 studies administering acupuncture exclusively on embryo transfer day (1 – 2 sessions, ≤30 min/session). Notably, >50% reported detrimental or null effects on pregnancy outcomes. We consider it to have a very limited impact on efficacy. Therefore, we excluded these 7 transfer-day acupuncture studies in sensitivity analyses to ensure evaluation of adequate treatment protocols. Acupuncture-assisted ART was found to be more favorable to increase the CPR and LBR compared with the control group, with a statistically significant difference in RR of 1.56 and 1.93, and 95% CI of 1.32-1.85 and 1.08-3.45, respectively (**Supplementary Figures 11**, **12**).

## 4 Discussion

Acupuncture is used as one of the most popular adjunctive complementary therapies for ART (56). Long-term acupuncture therapy enables patients to maintain stable mood and psychological well-being throughout ART. Mechanistic studies indicate that acupuncture can regulate endogenous modulation systems—including the sympathetic nervous system, endocrine system, and neuroendocrine pathways—to induce regular ovulation in women with polycystic ovary syndrome (57, 58) Research demonstrates that both state anxiety and trait anxiety are significantly associated with reduced clinical pregnancy rates following IVF treatment (59). During IVF-ET procedures, anxiety and depressive states in infertile women may disrupt maternal-fetal immune homeostasis and endocrine balance through the psychoneuroimmunoendocrine network. As a traditional Chinese therapeutic modality, acupuncture has been validated by multiple randomized controlled trials to mitigate infertility-related stress and anxiety while enhancing self-efficacy. These effects collectively contribute to significantly improved clinical pregnancy rates and live birth rates following ART treatment (13, 45).

In this study, we analyzed RCTs on the effect of acupuncture in improving ART pregnancy outcomes. We found that acupuncture was associated with higher clinical pregnancy rates (CPR) and live birth rates (LBR) compared to controls. These results are consistent with the findings of previous systematic studies (22, 60). Recent systematic reviews of adjuvant acupuncture for ART have pooled heterogeneous trials, without further analyzing the impact of different parameters of acupuncture protocol on the effectiveness (61). On this basis, we attempted to conduct further in-depth analyses of the effects of intervention timing, duration, total number of sessions, and frequency on the efficacy of the acupuncture intervention protocol by means of net meta-analysis.

The timing of acupuncture interventions is now the most complex and controversial factor in clinical implementation. The results of our study showed that the efficacy of adjuvant acupuncture on CPR was embryo culture period > ovarian stimulation period > ART preparation period > sham acupuncture > transfer day > no adjuvant treatment. In other words, better results were achieved by initiating acupuncture treatment early than by acupuncture only on transfer day. This result is consistent with previous systematic evaluations. When acupuncture was performed during controlled ovarian stimulation (COH), pregnancy outcomes in IVF were significantly better than in the control group when compared to other acupuncture times (CPR: OR 1.71, 95% CI 1.27-2.29, p = 0.0004; LBR: OR 2.41, 95% CI 1.54-3.78, p = 0.0001; BPR: OR 1.50, 95% CI 1.02-2.20, p = 0.04; OPR: OR 1.88, 95% CI 1.06-3.34, p = 0.03) (62). In addition, early assisted reproduction facilities often recommend a “day-of-transfer acupuncture” protocol to women undergoing IVF. This was acupuncture performed 25 minutes before and after ET according to the “Paulus protocol” (54). This acupuncture protocol seems to have become the standard in reproductive facilities, although there was only one successful randomized controlled trial at the time (26). The study we included, in which Craig, So, Andersen and Moy all followed such an acupuncture protocol, yielded negative results. They concluded that acupuncture does not improve clinical pregnancy rates in IVF-ET{sp} (42, 49–51){/sp}. We observed that trials advocating embryo transfer-day acupuncture were predominantly conducted in North America, South America, and Europe. This geographical distribution likely stems from divergences in cultural perceptions, healthcare systems, and research methodologies among nations, leading to marked variations in therapeutic approaches for the same condition. Therefore, we excluded eight papers on acupuncture on the transfer day for sensitivity analysis. The results showed that acupuncture was beneficial in increasing the CPR and LBR in IVF-ET. The results of this sensitivity analysis were generally consistent with the results of our net meta-analysis. Intervention of acupuncture treatment starting in the ART preparation period and ovarian stimulation period would have better efficacy compared with acupuncture on the transfer day. In addition, the results of Westergaard’s trial showed that early pregnancy miscarriage was more likely to occur when acupuncture treatment was received within 2 days of ET (47). This is one of the reasons why acupuncture is not recommended before or after the transfer day.

The total number of treatment sessions (N) and the total duration of treatment (D) are also key factors affecting the efficacy of acupuncture. Our analysis demonstrated that the longer the acupuncture duration (≥3 months) and the higher the number of acupuncture sessions (≥20 sessions), the better the CPR was improved. Yang’s results reported that in patients with premature ovarian insufficiency, exceeding 24 acupuncture treatments enhanced sinusoidal follicle counts (16). Other research indicated that the total number of acupuncture is an independent predictor of acupuncture’s impact on pregnancy outcomes, particularly in patients exhibiting ovarian hyporesponsiveness. Protocols administering >12 sessions yielded superior CPR compared to ≤12 sessions (47). Of course, many of the N and the D will consume more of the patient’s time and more of the patient’s medical resources. How to choose the right N and D based on the patient’s condition will be the key to the success of ART.

Frequency is a crucial factor influencing treatment efficacy. Among the included studies, acupuncture frequencies ranged from once to seven times weekly. However, only one study documented a once-weekly regimen, similarly, just one study implemented a twice-weekly protocol. For higher frequencies, five sessions weekly was reported in two studies, and seven sessions weekly was likewise documented in two studies. Due to the limited number of studies at these frequencies, we used three times a week as a cutoff point for analysis. The results of the systematic evaluation showed that the CPR of acupuncture-assisted intervention for ART had RR values of 1.46, 1.54, and 1.69 for high-frequency (greater than 3 times per week), medium-frequency (approximately equal to 3 times per week), and low-frequency (less than 3 times per week) treatments, respectively, with 95% CIs ranging from 1.03 to 2.06, 1.19 to 1.99, and 0.89 to 3.22, respectively. Our study did not conclude that higher frequency leads to better therapeutic outcomes. One literature analysis mentioned that the frequency of acupuncture treatment for dysmenorrhea was mostly once a day. If the patient had severe dysmenorrhea, the treatment could be given twice a day to relieve the pain symptoms. If the patient’s condition is stable, she can still be treated once every other day (63). An RCT showed that the long-term efficacy of electroacupuncture in improving perimenopausal symptoms was related to the frequency of acupuncture, and the long-term efficacy of 3 times a week was better than 1 time a week (64). A study showed that 3 times a week of electroacupuncture treatment is the optimal treatment duration to maintain the continuity efficacy for perimenopausal symptoms (65). Therefore, we believe that the determination of acupuncture frequency as an adjunct to ART should be based on a comprehensive assessment of factors such as the patient’s baseline pregnancy rate, the patient’s acceptance of the treatment, and the feasibility of the therapy.

Previous systematic reviews of acupuncture in ART have primarily concentrated on IVF-ET. Yet, recent technological advancements have expanded ART methodologies beyond conventional IVF-ET. According to the latest research report from the International Committee for Monitoring Assisted Reproductive Technologies indicates a steady increase in the utilization of ICSI, SET, and FET (66). Consequently, our study encompassed patients undergoing IVF-ET, as well as those who received ICSI and FET. Critical analysis of prior systematic reviews revealed substantial outcome heterogeneity: while some trials reported positive clinical outcomes (67), conversely others documented null effects (68). The reasons for the aforementioned situation may be due to the significant differences in the baseline conditions of the subjects. The baseline conditions of the subjects greatly influence the efficacy of ART interventions. Systematic review results indicate that trials with lower baseline pregnancy rates show a greater effect of adjunctive acupuncture (RR of 1.53, 95% CI of 1.28-1.84; 7 trials; 1,732 participants), while trials with higher baseline pregnancy rates show a smaller effect (RR of 0.90, 95% CI of 0.80-1.01; 9 trials; 2,289 participants) (69). A key limitation is that none of the 22 included studies reported baseline pregnancy rates, precluding subgroup analysis of this critical effect modifier. Although 13 studies (59%) documented causes of infertility in baseline characteristics (with no significant intergroup differences), this cannot substitute for direct quantification of baseline pregnancy probability. This gap necessitates future studies to standardize reporting of baseline prognostic factors.

Despite the detailed analyses performed in this analysis, such as sensitivity analyses, certain limitations remain. For example, we did not analyze the effect of combination treatments such as moxibustion, cupping, ear acupressure, and transcutaneous acupoint stimulation. A systematic review has shown that ear acupressure, moxibustion, and transcutaneous electrical acupoint stimulation have certain therapeutic effects on the pregnancy outcomes of ART (70). Similar to EA, moxibustion represents another traditional Chinese therapy that stimulates acupoints and meridians through the combined action of thermal energy, near-infrared radiation, and bioactive components in moxa smoke (71). Previous studies demonstrate that moxibustion enhances fertility in patients with diminished ovarian reserve by modulating reproductive pathways via multiple signaling mechanisms and molecular targets{sp} (72–74){/sp}. This deliberate exclusion narrows the scope of clinical applicability in critical dimensions, hence only 22 articles were included in the literature review. Secondly, for particular outcomes with limited available data—notably fertilization rate (reported in n=4 studies) and high-quality embryo rate (n=3 studies)—the small number of investigations constrains statistical power. Thirdly, there may be publication bias or small sample effect in this analysis. The number of literature corresponding to acupuncture treatment in embryo culture period is small, and the efficacy of acupuncture in this period still needs to be further verified in the future. In addition, some studies were heterogeneous. There were 14 studies with “some concerns” in terms of study quality, which increased the risk of bias. Finally, we have observed that some studies on acupuncture for improving ART pregnancy outcomes currently suffer from less than ideal research designs. It is hoped that future research can employ standardized research protocols to yield higher-quality evidence-based basis.

## 5 Conclusions

Adjunctive acupuncture during ART can enhance CPR, LBR, embryo quality, and fertilization rate. The most effective time of acupuncture treatment appears to be embryo culture period, during Ovarian stimulation period or ART preparation period. In addition, longer duration of treatment (≥3 months) and higher total treatment numbers (≥20 sessions) may lead to better improvements in pregnancy outcomes. However, due to the heterogeneity of studies included and the potential for publication bias, these findings warrant further validation through high-quality research.
