# Supplementary material for: Different effectiveness of acupuncture treatment schedule on ART pregnancy outcomes: a systematic review and network meta-analysis
Source: Front Endocrinol (Lausanne). 2025 Sep 5;16:1602710. doi: 10.3389/fendo.2025.1602710 (PMC12446238; doi:10.3389/fendo.2025.1602710)
Supplement: Supplementary file 1 [file DataSheet1.pdf]

Contents

Supplementary Table 1 Characteristics of included trials..1

Supplementary Figure 1.Risk of bias of included trials..5

Supplementary Figure 2.Risk of bias summary of included trials..5

Supplementary Figure 3. Network of different acupuncture intervention protocols assisting ART to improve CPR...6

Supplement Table 2. Estimated Global Inconsistency in Networks..6

Supplement Table 3. Estimated Local Inconsistency for each pairwise comparison (side splitting).........7

Supplementary Figure 4. Forest of Consistency Diagram (timing of different acupuncture interventions)...8

Supplementary Figure 5. Consistency forest plot (different acupuncture treatment durations).................8

Supplementary Figure 6. Forest plot of consistency (total number of different acupuncture treatments).9

Supplement Table 4. Results of Net Meta-analysis comparing the effect of T on pregnancy outcome.....9

Supplement Table 5. Results of Net Meta-analysis comparing the effect of D on pregnancy outcomes..10

Supplement Table 6. Results of Net Meta-analysis comparing the effect of N on pregnancy outcomes..10

Supplement Figure 7.Ranking of optimal probability of pregnancy outcome in ART patients with different acupuncture intervention protocols...10

Supplementary Figure 8. Funnel plot (timing of different acupuncture interventions)............................11

Supplementary Figure 9. Leakage chart (different acupuncture treatment durations).............................11

Supplementary Figure 10. Leakage chart (total number of different acupuncture treatments).................12

Supplementary Figure 11. Forest plot of CPR in acupuncture group and control group after sensitivity analysis...12

Supplementary Figure 12. Forest plot of LBR in acupuncture group and control group after sensitivity analysis...12

The references of the final included clinical studies..13

Supplementary Table 1 | Characteristics of included trials.

References	Year	Country	N (T/C)	Age (T/C, years)	Infertility time*a(T/C)	BMI(T/C, Kg/m²)	Acupuncture intervention therapy Method	Acupuncture intervention therapy Timing	Control group	Outcomes
Xie et al.[1]	2023	China	33/32	T: 34.21±3.02<br>C:35.53±3.42	T: 52.30±25.73<br>C:52.03±50.77	T: 22.3（20.7，23.8）<br>C:21.5（19.8，24.3）	AC	**Start time:** 2 menstrual cycles before OPU(except menstrual period)<br>**Frequency:** 3 times a week(last 30min*b)<br>**Duration:** 2 menstrual cycle	No adjuvant treatment	①③⑤
Guo et al.[2]	2022	China	23/21	T: 34.57±2.84<br>C: 33.70±3.55	T: 4.87±1.59<br>C: 4.17±1.60	T: 23.12±2.56<br>C: 22.93±2.36	EA	**Start time:** days 2 to 5 of your menstrual cycle<br>**Frequency:** every other day(last 20min)<br>**Duration:** 1 menstrual cycle to the day of OPU	No adjuvant treatment	①④
Shen et al.[3]	2022	China	32/33	T: 35.0±6.0<br>C: 37.0±7.0	T: 5.9±4.3<br>C: 4.5±4.4	T: 22.9±3.8<br>C: 22.7±3.4	EA	**Start time:** 3 menstrual cycles before embryo transfer(except menstrual period)<br>**Frequency:** 3 times a week(last 30min)<br>**Duration:** 3 menstrual cycle, the day of OPU to 1 day before ET	No adjuvant treatment	①②
Wu et al.[4]	2021	China	50/50	T: 31.00±3.00<br>C: 31.00±3.00	T: 3.62±1.97<br>C: 3.71±2.20	T: 22.56±3.31<br>C: 22.76±3.65	AC	**Start time:** 3 menstrual cycles before OPU(except menstrual period)<br>**Frequency:** every other day(last 30min)<br>**Duration:** 3 menstrual cycle	No adjuvant treatment	①④⑤
Zhong et al.[5]	2021	China	30/31	T: 36.52±2.11<br>C: 36.19±1.95	T: 3.96±1.22<br>C: 3.55±1.82	T: 26.24±2.12<br>C: 25.35±2.76	EA	**Start time:** 1 menstrual cycles before IVF-ET(except menstrual period)<br>**Frequency:** 3 times a week(last 30min)<br>**Duration:** 1 menstrual cycles	No adjuvant treatment	①③④
Xiang et al.[6]	2021	China	33/33	T: 39.82±5.41<br>C: 38.67±5.36	T: 1.45±0.96<br>C: 1.38±2.27	T: 22.42±7.24<br>C: 23.13±7.13	EA	**Start time:** 1 menstrual cycles before COH(except menstrual period)<br>**Frequency:** 2 times a week(last 30min)<br>**Duration:** 1 menstrual cycle to trigger Day	Placebo acupuncture	①⑤

Supplementary Table 1 | Characteristics of included trials(Continued).

References	Year	Country	N (T/C)	Age (T/C, years)	Infertility time*(T/C)	BMI(T/C, Kg/m²)	Acupuncture intervention therapy		Control group	Outcomes
							Method	Timing		
Dehghani et al.[7]	2020	Iran	62/62	T: 32.9 ± 4.8<br>C: 31.5 ± 5.4	T: 5.1 ± 3.1<br>C: 5.4 ± 4	T: 25.2 ± 3.8<br>C: 26.3 ± 3.9	AC	**Start time:** 25 minutes before and after ET<br>**Frequency:** two times in total(last 25min)<br>**Duration:** before and after ET	No adjuvant treatment	①
Guven et al.[8]	2020	Turkey	36/36	T: 30.3 ± 3.4<br>C: 31.5 ± 4	-	T: 24.4 ± 3.0<br>C: 23.3 ± 1.9	AC	**Start time:** 1 week before ET<br>**Frequency:** three times in total(last 30min)<br>**Duration:** 1 week before ET, 30 minutes before and after ET	No adjuvant treatment	①②
Altutunji et al.[9]	2019	China	33/69	T: 20-40[*c]<br>C: 20-40	-	-	AC	**Start time:** the third day of your menstrual cycle<br>**Frequency:** every day(last 30-40min)<br>**Duration:** the third day of your menstrual cycle to 14 days after ET	No adjuvant treatment	①③
Smith et al.[10]	2018	Australian	304/307	T: 35.4 (4.3)[*d]<br>C: 35.5 (4.3)	-	T: 25.8 (5.5) [*d]<br>C: 26.0 (5.8)	AC	**Start time:** days 6 to 8 of ovarian stimulation<br>**Frequency:** two times in total(last 25min)<br>**Duration:** days 6 to 8 of ovarian stimulation and the day of ET	Sham acupuncture	①②
Craig et al.[11]	2014	America	57/56	T: 33.4 (5.8)[*d]<br>C: 32.8 (5.9)	-	-	AC	**Start time:** 25min treatment session before ET<br>**Frequency:** one times in total(last 25min)<br>**Duration:** 25min treatment session both before and after ET	No adjuvant treatment	①②
Rashidi et al.[12]	2013	Iran	31/31	T: 31.03±4.82<br>C: 32.10±4.68	T: 9.09±4.65<br>C: 9.41±4.93	T: 27.83±4.61<br>C: 26.10±4.15	AC	**Start time:** on the 21st day of the previous cycle<br>**Frequency:** four times in total(last 30min)<br>**Duration:** on the 21st day of the previous cycle, 2 days before OPU, and immediately before and after ET	No adjuvant treatment	①

Supplementary Table 1 | Characteristics of included trials(Continued).

References	Year	Country	N (T/C)	Age (T/C, years)	Infertility time*(T/C)	BMI(T/C, Kg/m^2)	Acupuncture intervention therapy		Control group	Outcomes
							Method	Timing		
Isoyama et al.[13]	2012	Brazil	22/21	T: 34.1±4.6<br>C: 34.3±4.6	T: 3.4±1.5<br>C: 4.7±2.8	-	AC	**Start time:** 1 month before ET<br>**Frequency:** once a week(last 25min)<br>**Duration:** Minimum 4 times, maximum 6 times. Last time after ET	Non-meridian point	①
Cui et al.[14]	2011	China	34/32	T: 29.29±3.66<br>C: 29.28±3.45	T: 4.00±2.62<br>C: 4.25±3.01	T: 24.24±4.13<br>C: 23.96±3.14	EA	**Start time:** 1 menstrual cycles before COH(except menstrual period)<br>**Frequency:** treatment for 5 days, rest for 1-2 days(last 30min)<br>**Duration:** 1 menstrual cycle to the day of OPU	No adjuvant treatment	①②③④⑤
Moy et al.[15]	2011	America	86/74	T: 33.30±0.31<br>C: 33.16±0.33	-	T: 24.77 ±1.051<br>C: 24.05±0.582	AC	**Start time:** before ET<br>**Frequency:** two times in total(last 25min)<br>**Duration:** before and after ET	Sham acupuncture	①
Madaschi et al.[16]	2010	Brazil	208 /208	T: 35.3±4.7<br>C: 34.6±4.6	-	T: 22.4 ± 3.8<br>C: 22.4 ± 2.9	AC	**Start time:** 25min immediately before ET<br>**Frequency:** two times in total(last 25min)<br>**Duration:** 25min before and immediately after ET	No adjuvant treatment	①②
Andersen et al.[17]	2010	Danish	314 /321	T: 31 (26, 37)<br>C: 31 (26, 36)	T: 2.5 (1.0,6.0)<br>C: 2.5 (1.0,5.0)	T: 22.5 (19, 31)<br>C: 22.5 (19, 33)	AC	**Start time:** 30 min immediately before ET<br>**Frequency:** two times in total(last 30min)<br>**Duration:** 30min before and immediately after ET	Placebo acupuncture	①②
Li et al.[18]	2009	China	23/20	T: 32.63±4.26<br>C: 32.48±4.45	T: 6.86±2.62<br>C: 6.75±3.01	T: 24.66±3.65<br>C: 24.13±3.34	EA	**Start time:** 1 menstrual cycles before COH(except menstrual period)<br>**Frequency:** treatment for 5 days, rest for 1-2 days(last 30min)<br>**Duration:** 1 menstrual cycle to the day of OPU	No adjuvant treatment	①③④⑤

Supplementary Table 1 | Characteristics of included trials(Continued).

References	Year	Country	N (T/C)	Age (T/C, years)	Infertility time*(T/C)	BMI(T/C, Kg/m^2)	Acupuncture intervention therapy		Control group	Outcomes
							Method	Timing		
Chen et al.[19]	2009	China	30/30	T: 34.33±2.71<br>C: 34.60±2.38	T: 6.26±4.31<br>C: 6.08±4.66	T: 22.53±1.70<br>C: 22.47±2.02	EA	**Start time:** 1 menstrual cycle before superovulation(except menstrual period)<br>**Frequency:** every day(last 30min)<br>**Duration:** 1 menstrual cycle and before OPU	No adjuvant treatment	①③④⑤
So et al.[20]	2009	China	185 /185	T:36.0(33-38)*e<br>C: 36.0(34-38)	T:4.0(2.0-6.0)*e<br>C: 4.0(2.5-7.0)	T: 21.6±2.1<br>C: 21.7±2.7	AC	**Start time:** 25min immediately before ET<br>**Frequency:** two times in total(last 25min)<br>**Duration:** 25min before and immediately after ET	Placebo acupuncture	①②
Smith et al.[21]	2006	Australia	107 /114	T: 35.9(4.7)*d<br>C: 36.1(4.8)	-	T: 25.4 (4.2)*d<br>C: 26.0 (5.6)	AC	**Start time:** on day 9 of stimulating injections<br>**Frequency:** three times in total(last 25min)<br>**Duration:** on day 9 of stimulating injections, before and after ET	Non-meridian point	①
Westergaard et al.[22]	2006	Denmark	91/87	T: 37(27-45)*e<br>C: 37(27-45)	T: 4 (1-10)*e<br>C: 4 (1-9)	T: 22 (18-34)*e<br>C: 23 (18-32)	AC	**Start time:** 25min immediately before ET<br>**Frequency:** two times in total(last 25min)<br>**Duration:** 25min before and immediately after ET	No adjuvant treatment	①

*a: Infertility time(months or years); *b: Duration of each session;*c: Patients aged 20-40 years;*d: mean (SD); *e：Data are given in median (interquartile range).

T, the treatment group; C, the control group; AC, acupuncture;EA, electro-acupuncture;COH, controlled ovarian hyperstimulation; ET, embryo transfer; OPU,ovum pick-up .

Outcomes: ①clinical pregnancy rate(CPR); ②live birth rate(LBR); ③rate of fertilization; ④number of retrieved oocytes; ⑤high-quality embryonic rate.

Supplementary Figure 1.Risk of bias of included trials.

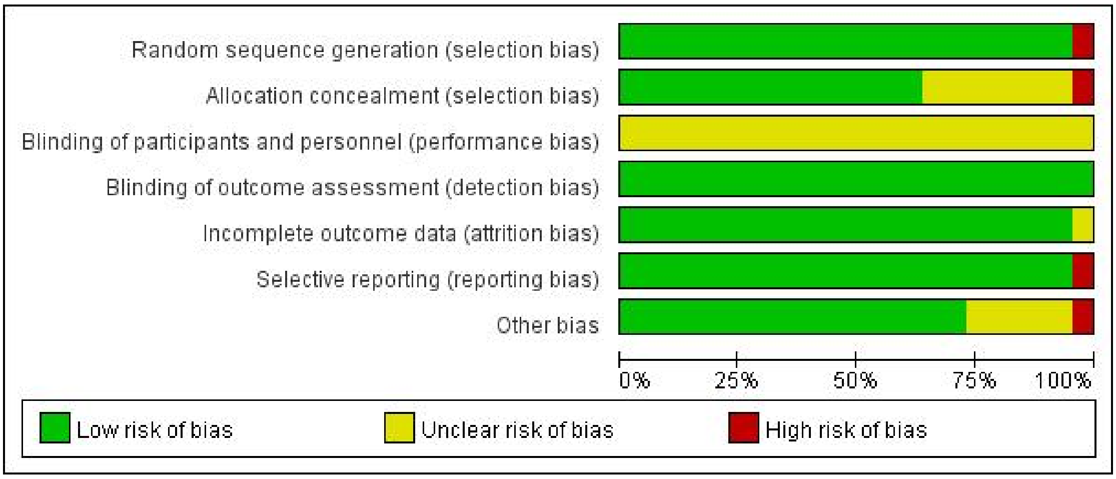

Supplementary Figure 2. Risk of bias summary of included trials.

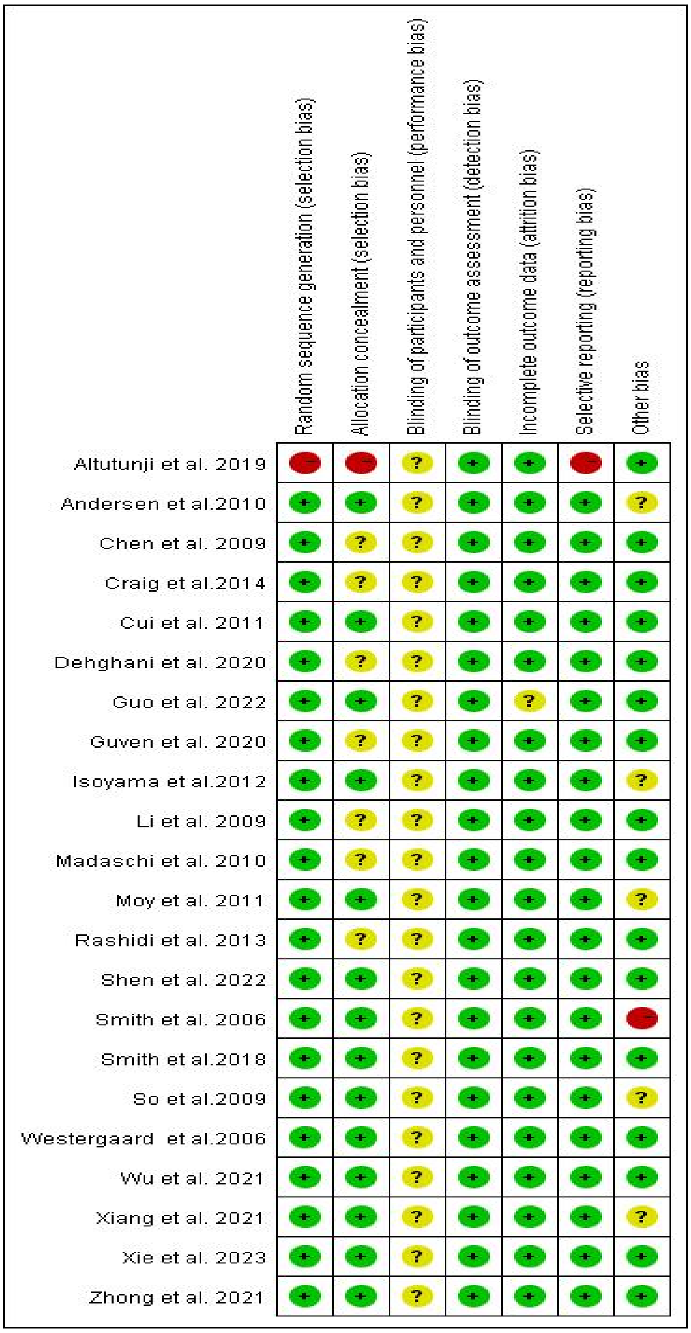

Supplementary Figure 3. Network of different acupuncture intervention protocols assisting ART to improve CPR.

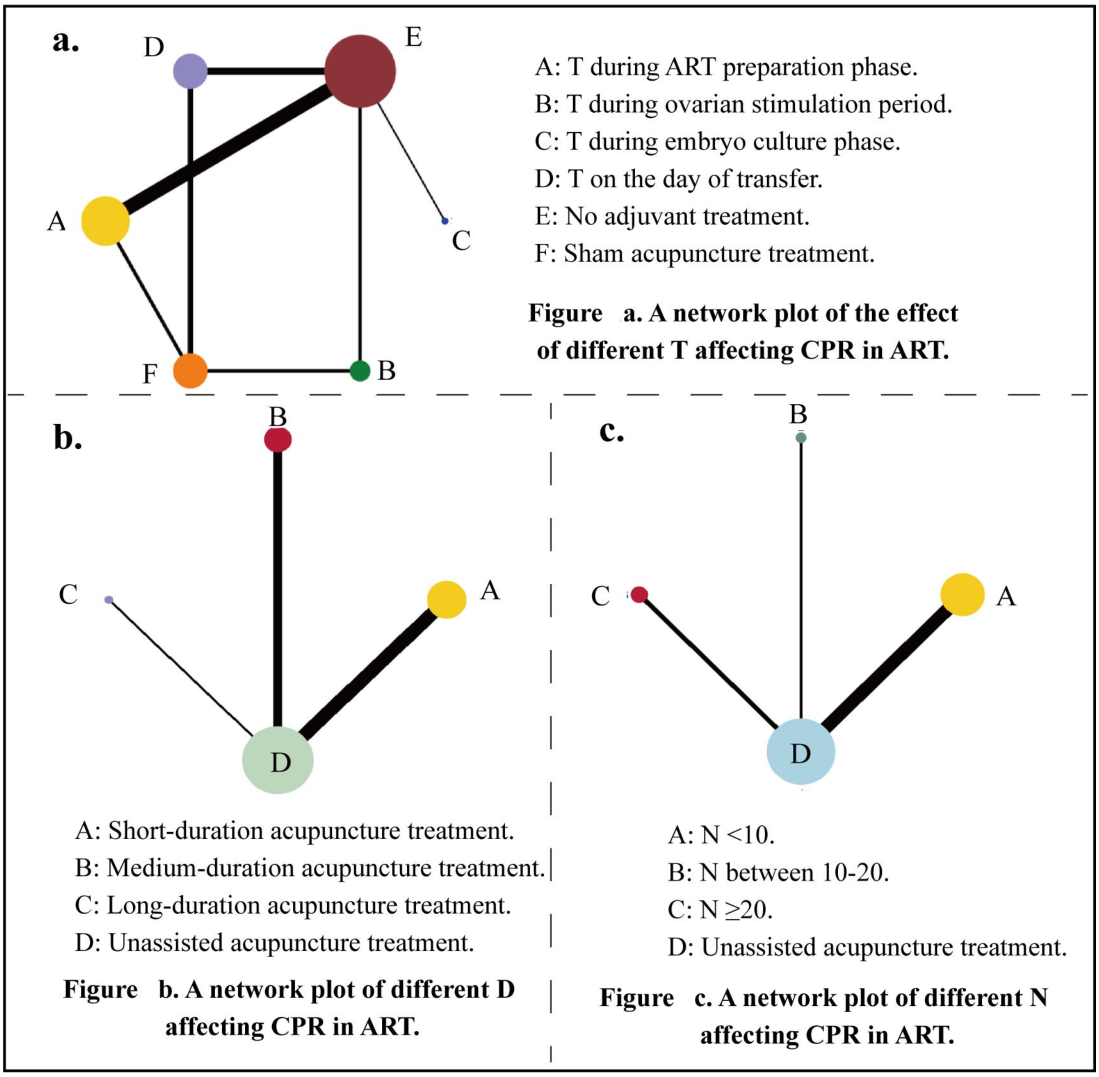

Figure a. A network plot of the effect of different T affecting CPR in ART.

Figure b. A network plot of different D affecting CPR in ART.

Figure c. A network plot of different N affecting CPR in ART.

T: time of starting acupuncture treatment, D: total duration of treatment, N: total number of treatment sessions.

Supplement Table 2. Estimated Global Inconsistency in Networks.

Outcome	Follow up	Chi square	Prob > chi2
CPR	Time of starting treatment	chi2 (2) = 1 .36	Prob > chi2 = 0.5057
	Total duration of treatment	chi2 (1) = 6.52	Prob > chi2 = 0.0107*
	Total number of treatment sessions	chi2 (1) = 2.81	Prob > chi2 = 0.0938

CPR: clinical pregnancy rate.

* Global consistency is tested here using the ‘design-by-interaction’ test that infers consistency across an entire treatment network, using a chi square test. A p value < 0.05 is taken to infer evidence of global inconsistency in the network.

Supplement Table 3. Estimated Local Inconsistency for each pairwise comparison (side splitting).

Supplement Table 3a. Time of starting treatment.

**Side**	**Direct**		**Indirect**		**Difference**		**tau**
	Coef.	Std.Err.	Coef.	Std.Err.	Coef.	Std.Err.	P>|z|
A-E	-0.3872707	0.1271586	-0.8598372	0.3849466	0.4725665	0.4055053	0.244
A-F	-0.5259144	0.3501959	-0.052686	0.2044507	-0.4732284	0.4056721	0.243
B-E	-0.5087163	0.3298875	-0.4924933	0.2379126	-0.016223	0.4066657	0.968
B-F	-0.2353877	0.1718103	-0.2516244	0.3685759	0.0162367	0.4066629	0.968
C-E*	-0.6505876	0.3130104	-0.7635662	65.03041	0.1129786	65.03178	0.999
D-E	-0.1978621	0.1367087	0.0862127	0.2956873	-0.2840749	0.3278764	0.386
D-F	0.1547379	0.1281986	-0.1294547	0.3017251	0.2841926	0.3279044	0.386

A: ART preparation period, B:ovarian stimulation period, C: embryo culture period, D: embryo transfer day, E: No adjuvant treatment, F: Sham acupuncture.

* All the evidence about these contrasts comes from the trials which directly compare them.

Supplement Table 3b. Total duration of treatment.

**Side**	**Direct**		**Indirect**		**Difference**		**tau**
	Coef.	Std.Err.	Coef.	Std.Err.	Coef.	Std.Err.	P>|z|
A-D*	-0.1202554	0.0936018	-0.2348182	12.66712	0.1145628	12.66746	0.993
B-D*	-0.3528523	0.1535296	-0.2240817	30.318	-0.1287706	30.31851	0.997
C-D*	-0.6262735	0.2452255	-0.1652575	46.09182	-0.461016	46.09288	0.992

A: short duration, B:medium duration, C: long duration, D: unassisted acupuncture treatment.

* All the evidence about these contrasts comes from the trials which directly compare them.

Supplement Table 3c. Total number of treatment sessions.

**Side**	**Direct**		**Indirect**		**Difference**		**tau**
	Coef.	Std.Err.	Coef.	Std.Err.	Coef.	Std.Err.	P>|z|
A-D*	-0.2196947	0.1070713	-0.1439939	12.77403	-0.0757008	12.77445	0.995
B-D*	-0.2322837	0.2414259	-0.4243452	42.92627	0.1920615	42.92709	0.996
C-D*	-0.3044548	0.1816699	-0.4335564	31.78712	0.1291016	31.78779	0.997

A: less than 10 sessions, B:between 10 and 20 sessions, C: more than or equal 20 sessions, D: unassisted acupuncture treatment.

* All the evidence about these contrasts comes from the trials which directly compare them.

Supplementary Figure 4. Forest of Consistency Diagram (timing of different acupuncture interventions).

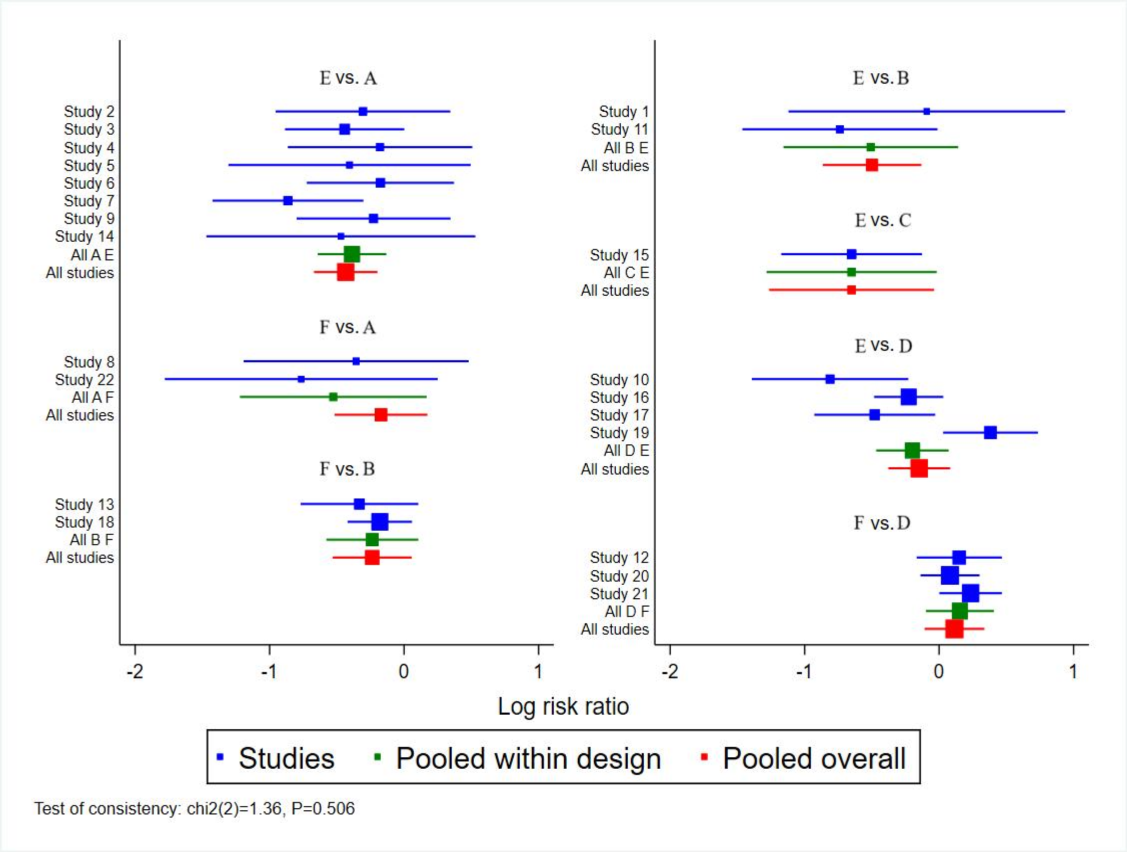

A: ART preparation period, B: ovarian stimulation period, C: embryo culture period, D: transfer day, E: no adjuvant treatment, F: sham acupuncture.

Supplementary Figure 5. Consistency forest plot (different acupuncture treatment durations).

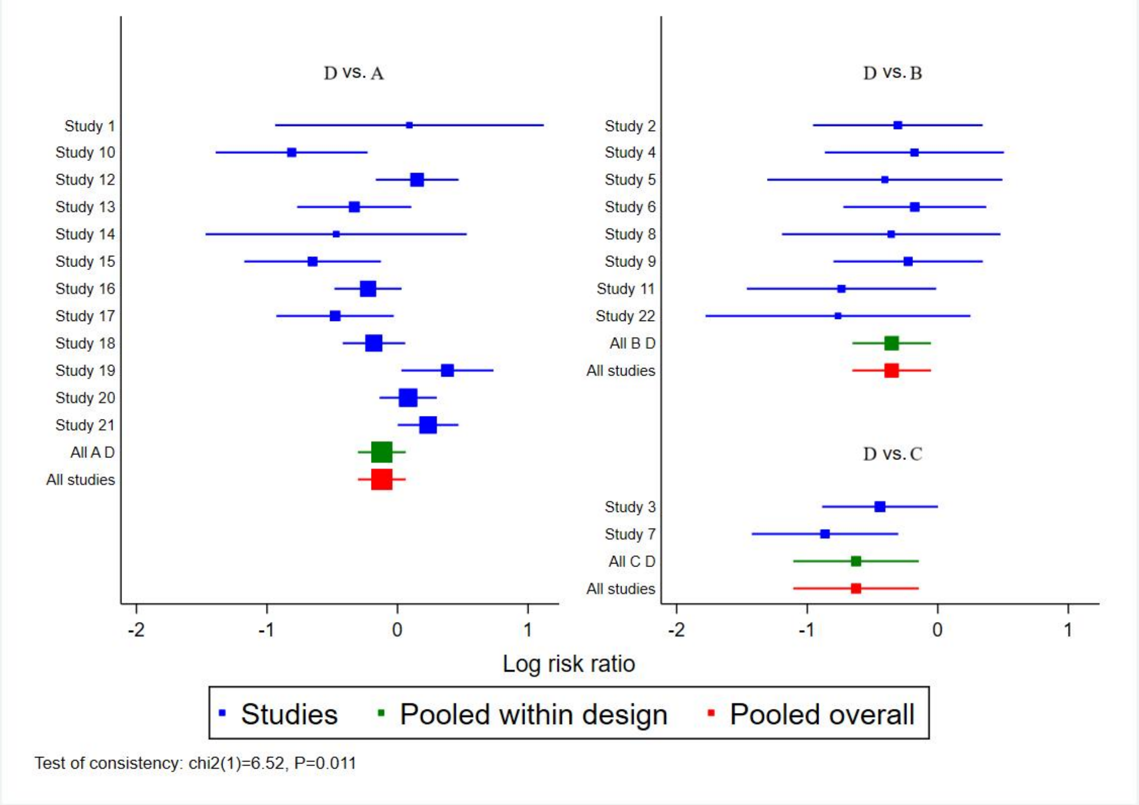

A: short duration(less than one menstrual cycle), B: medium duration (greater than one month and less than 2 months), C: long duration(greater than or equal to 2 menstrual cycles), D: non acupuncture treatment.

Supplementary Figure 6. Forest plot of consistency (total number of different acupuncture treatments).

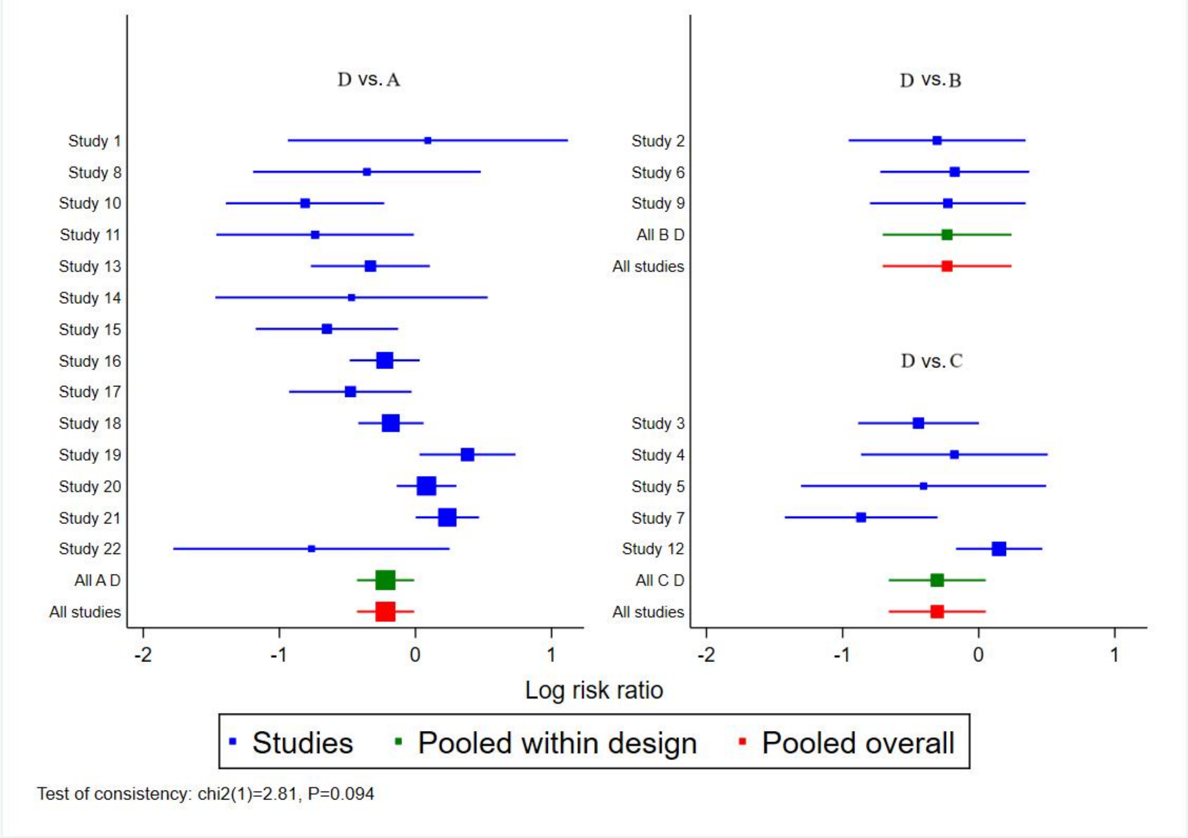

A: less than 10 sessions, B: between 10 and 20 sessions, C: more than or equal 20 sessions, D: 0 sessions at real acupuncture points(non acupuncture treatment).

Supplement Table 4. Results of Net Meta-analysis comparing the effect of T on pregnancy outcome.

Embryo culture period					
0.15 (-0.56,0.87)	Ovarian stimulation period				
0.22 (-0.44,0.87)	0.06 (-0.35,0.48)	ART preparation period			
0.39 (-0.29,1.07)	0.24 (-0.06,0.53)	0.17 (-0.17,0.52)	Sham acupuncture		
0.50 (-0.15,1.16)	0.35 (0.01,0.70)	0.29 (-0.03,0.60)	0.11 (-0.11,0.34)	Embryo transfer day	
0.65 (0.04,1.26)	0.50 (0.13,0.87)	0.43 (0.20,0.67)	0.26 (-0.03,0.55)	0.15 (-0.08,0.38)	No adjuvant treatment

T: time of starting acupuncture treatment.

Supplement Table 5. Results of Net Meta-analysis comparing the effect of D on pregnancy outcomes.

Long duration			
0.27 (-0.29,0.84)	Medium duration		
0.51 (-0.01,1.02)	0.23 (-0.12,0.58)	Short duration	
0.63 (0.15,1.11)	0.35 (0.05,0.65)	0.12 (-0.06,0.30)	Non acupuncture

D: total duration of treatment

Supplement Table 6. Results of Net Meta-analysis comparing the effect of N on pregnancy outcomes.

≥20 times			
0.31 (-0.26,0.87)	10-20 times		
0.39 (-0.01,0.79)	0.08 (-0.39,0.55)	＜10 times	
0.54 (0.18,0.89)	0.23 (-0.20,0.67)	0.15 (-0.03,0.33)	0 times

N: total number of treatment sessions.

Supplement Figure 7.Ranking of optimal probability of pregnancy outcome in ART patients with different acupuncture intervention protocols.

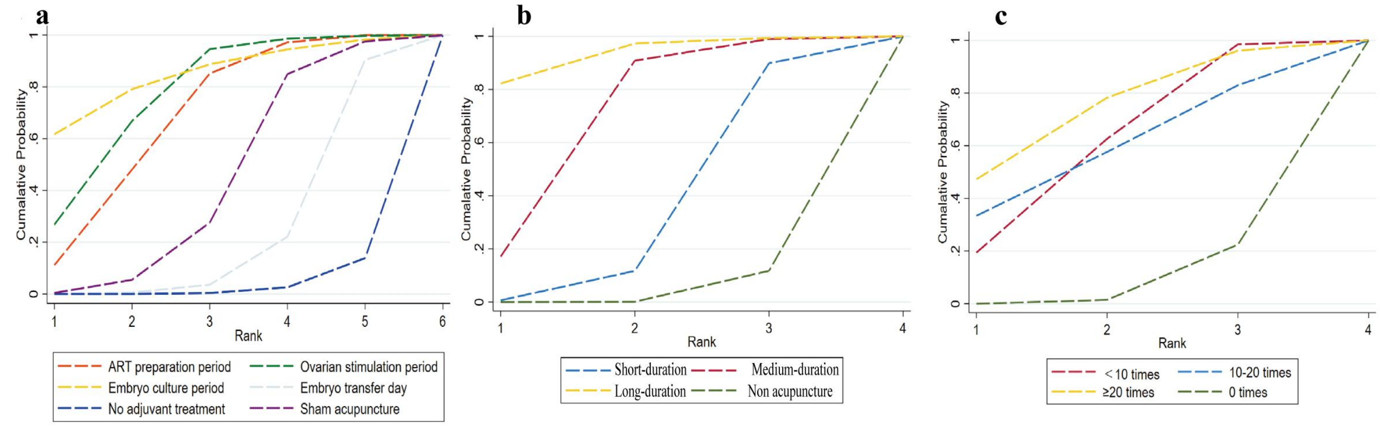

Figure a. Time of starting acupuncture treatment

Figure b. Total duration of treatment

Figure c. Total number of treatment sessions

Supplementary Figure 8. Funnel plot (timing of different acupuncture interventions).

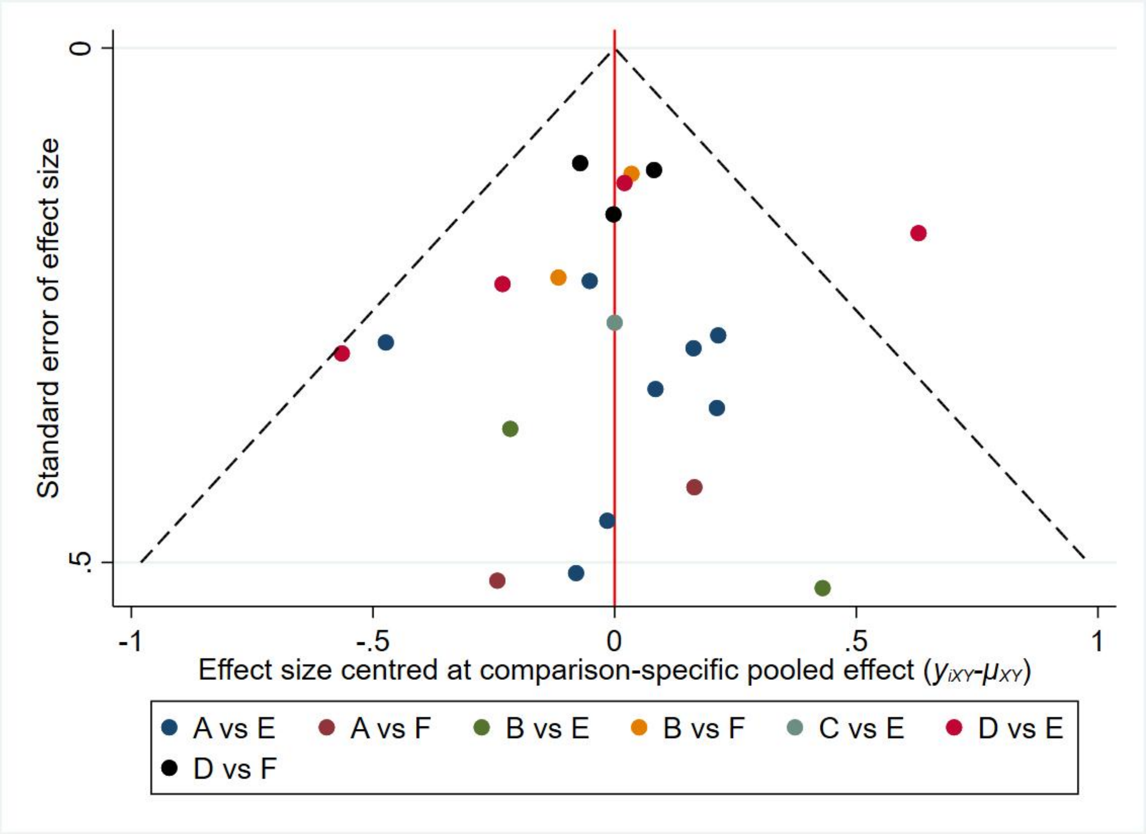

A: ART preparation period, B: ovarian stimulation period, C: embryo culture period, D: transfer day, E: no adjuvant treatment, F: sham acupuncture.

Supplementary Figure 9. Leakage chart (different acupuncture treatment durations).

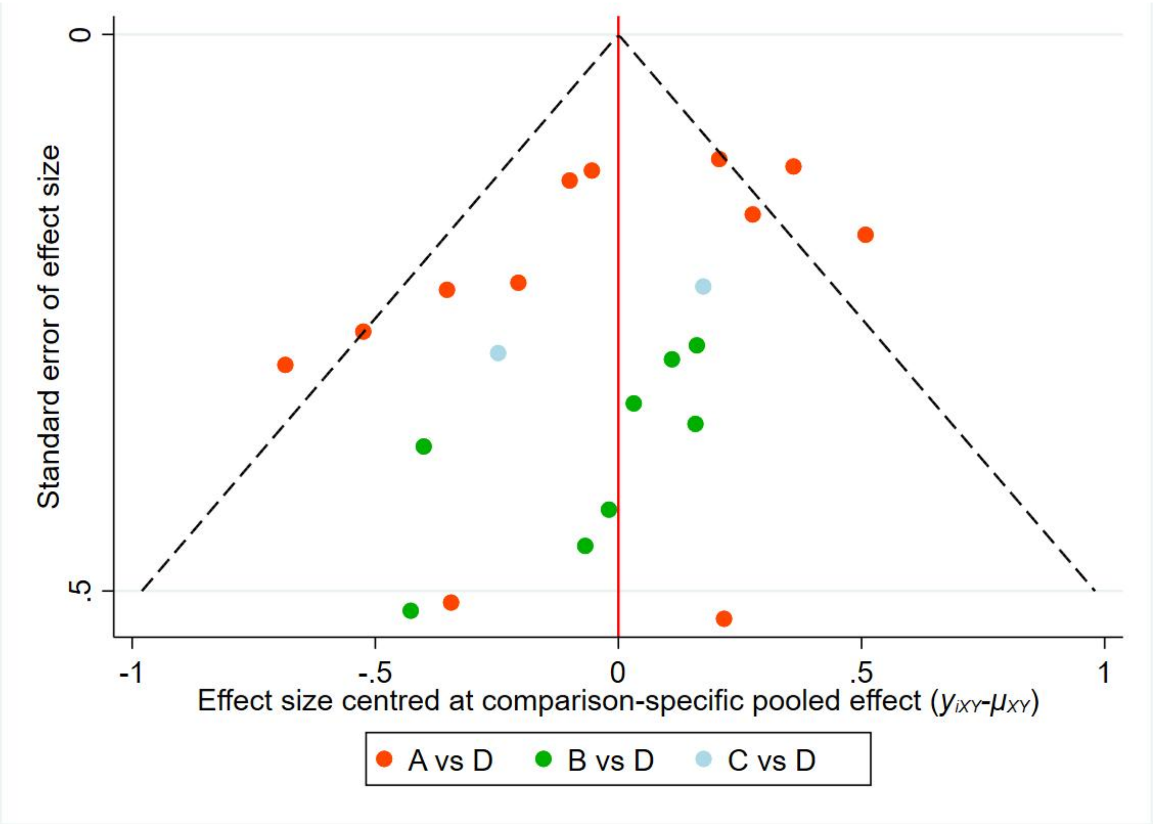

A: short duration(less than one menstrual cycle), B: medium duration (greater than one month and less than 2 months), C: long duration(greater than or equal to 2 menstrual cycles), D: non acupuncture treatment.

Supplementary Figure 10. Leakage chart (total number of different acupuncture treatments).

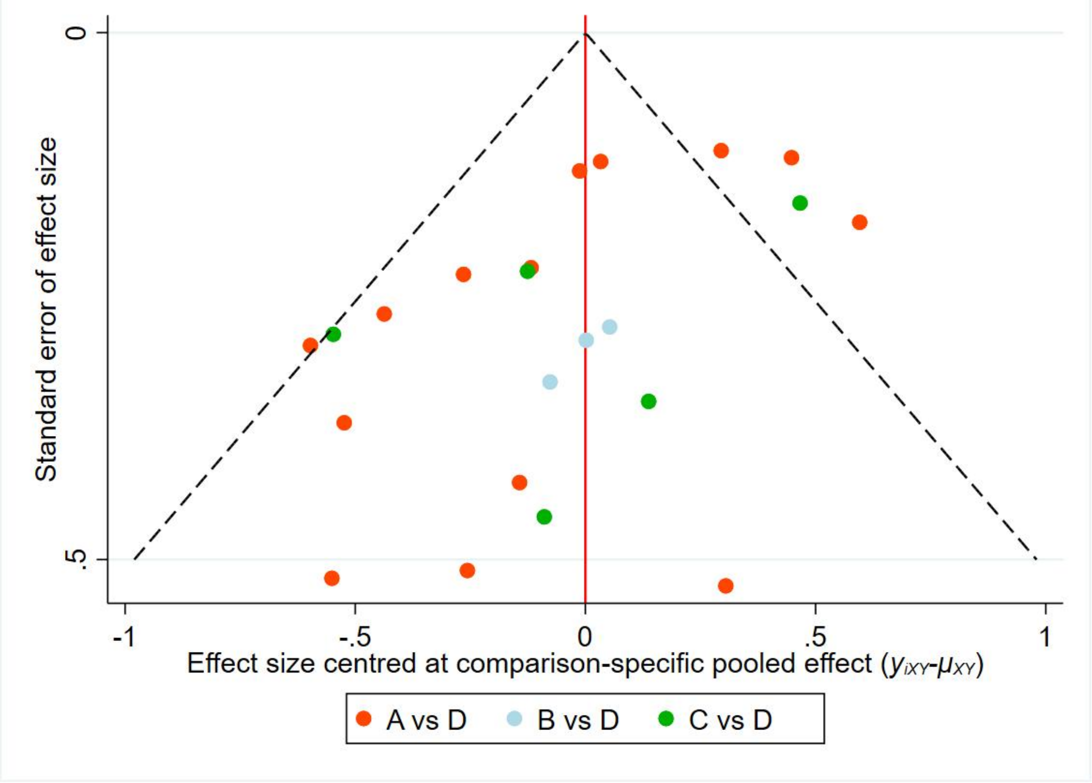

A: less than 10 sessions, B: between 10 and 20 sessions, C: more than or equal 20 sessions, D: 0 sessions at real acupuncture points(non acupuncture treatment).

Supplementary Figure 11. Forest plot of CPR in acupuncture group and control group after sensitivity analysis.

Study or Subgroup	Acupuncture Events	Acupuncture Total	Control Events	Control Total	Weight	Risk Ratio M-H, Fixed, 95% CI
Altutunji et al. 2019	11	33	11	69	5.0%	2.09 [1.01, 4.32]
Chen et al. 2009	9	20	6	30	3.4%	2.25 [0.95, 5.34]
Cui et al. 2011	16	34	12	32	8.7%	1.25 [0.71, 2.22]
Guo et al. 2022	6	23	5	21	3.7%	1.10 [0.39, 3.07]
Guven et al. 2020	23	36	12	36	8.4%	1.92 [1.14, 3.23]
Isoyama et al.2012	9	22	4	21	2.9%	2.15 [0.78, 5.92]
Li et al. 2009	11	23	8	20	6.0%	1.20 [0.60, 2.37]
Rashidi et al. 2013	8	31	5	31	3.5%	1.60 [0.59, 4.35]
Shen et al. 2022	23	32	10	33	6.9%	2.37 [1.35, 4.16]
Smith et al. 2006	34	107	26	114	17.7%	1.39 [0.90, 2.16]
Wu et al. 2021	28	50	18	50	12.7%	1.56 [1.00, 2.42]
Xiang et al. 2021	10	33	7	33	4.9%	1.43 [0.62, 3.30]
Xie et al. 2023	14	33	10	32	7.1%	1.36 [0.71, 2.60]
Zhong et al. 2021	15	30	13	31	9.0%	1.19 [0.69, 2.06]
**Total (95% CI)**		**507**		**553**	**100.0%**	**1.56 [1.32, 1.85]**
Total events	217		147			

Heterogeneity: Chi² = 7.43, df = 13 (P = 0.88); I² = 0%
Test for overall effect: Z = 5.23 (P < 0.00001)

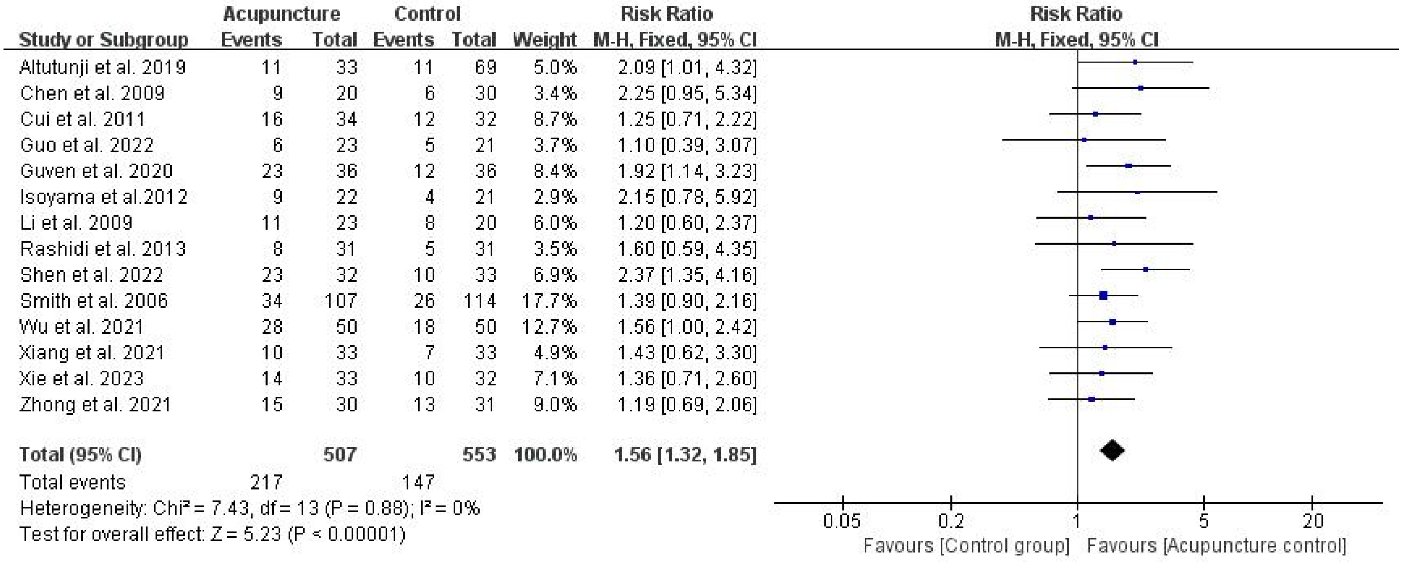

Supplementary Figure 12. Forest plot of LBR in acupuncture group and control group after sensitivity analysis.

Study or Subgroup	Acupuncture Events	Acupuncture Total	Control Events	Control Total	Weight	Risk Ratio M-H, Random, 95% CI
Cui et al. 2011	29	34	20	32	44.0%	1.36 [1.01, 1.85]
Guven et al. 2020	19	36	10	36	32.2%	1.90 [1.03, 3.50]
Shen et al. 2022	18	32	5	33	23.8%	3.71 [1.57, 8.80]
**Total (95% CI)**		**102**		**101**	**100.0%**	**1.93 [1.08, 3.45]**
Total events	66		35			

Heterogeneity: Tau² = 0.18; Chi² = 6.31, df = 2 (P = 0.04); I² = 68%
Test for overall effect: Z = 2.21 (P = 0.03)

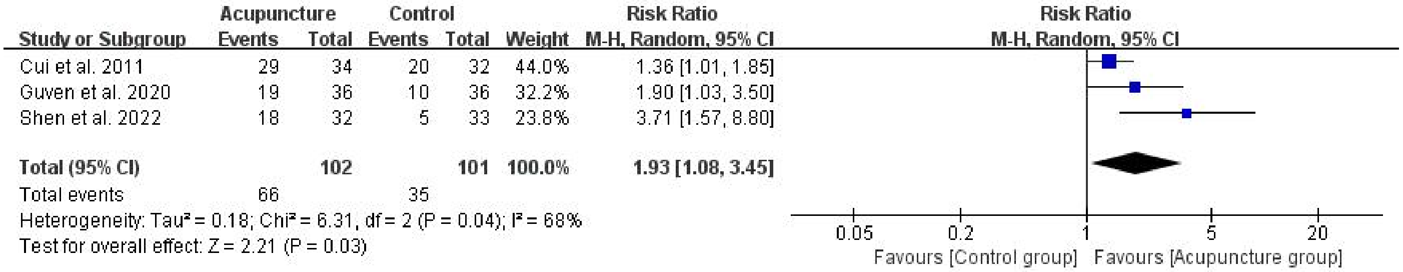

The references of the final included clinical studies.

[1] XIE Qingling, LAN Ying, ZHU Xinyun et al. Effects of acupuncture on embryo quality and clinical pregnancy rate in patients with poor ovarian response to in vitro fertilization-embryo transfer [J]. China Journal of Traditional Chinese Medicine and Pharmacy, 2023, 38 (01): 419-422.

[2] GUO Yanjing, ZHAO Fang. Impact of Acupuncture on the In-vitro Fertilization and Embryo Transfer Outcomes in Patients with Premature Ovarian Insufficiency [J], Shanghai Journal of Acupuncture and Moxibustion. 2022,41(11):1084-1088.

[3] Shen J, Gao YL, Lu G, Chen L, Cheng J, Xia YB. Effect of electroacupuncture on endometrial receptivity and IVF-ET pregnancy outcomes in patients with diminished ovarian reserve [J]. Zhongguo Zhen Jiu. 2022;42(8):879-883. doi:10.13703/j.0255-2930.20210901-k0002.

[4] WU Jiaman, ZHUO Yuanyuan, TANG Meng et al.Effect of Tiao Ren Tong Du Needling Method on Pregnancy Outcome After IVF-ET for Infertility Due to Polycystic Ovary Syndrome [J]. Shanghai Journal of Acupuncture and Moxibustion, 2021,40(05):565-570.DOI:10.13460/j.issn.1005-0957.2020.13.1069.

[5] ZHONG Xiufang， REN Chunxia， CHI Lingkan et al. Effects of electroacupuncture intervention on endometrial receptivity and pregnancy outcome in patients undergoing IVF-ET with syndrome of kidney deficiency and phlegm stasis [J]. Academic Journal of Shanghai University of Traditional Chinese Medicine, 2021,35(02):28-31+37.DOI:10.16306/j.1008-861x.2021.02.006.

[6] XIANG Shan, HAO Hao, LIAN Fang.Study on Effect of Electro-acupuncture on Oocyte Quality in Elderly Infertile IVF Patients [J]. Shandong Journal of Traditional Chinese Medicine, 2021,40(03):284-288.DOI:10.16295/j.cnki.0257-358x.2021.03.013.

[7] Dehghani AS, Homayouni K, Kanannejad Z, Kanannejad Z. The effect of acupuncture on the day of embryo transfer on the in vitro fertilization outcomes: An RCT. Int J Reprod Biomed. 2020;18(3):209-214. Published 2020 Mar 29. doi:10.18502/ijrm.v18i3.6719.

[8] Guven PG, Cayir Y, Borekci B. Effectiveness of acupuncture on pregnancy success rates for women undergoing in vitro fertilization: A randomized controlled trial. Taiwan J Obstet Gynecol. 2020;59(2):282-286. doi:10.1016/j.tjog.2020.01.018.

[9] Altutunji AZ, Liu L, Cai J, Wang Z, Gao Y. The effect of acupuncture on anti-mullerian hormone and assisted reproduction outcome in Polycystic Ovary Syndrome patients undergoing in vitro fertilization. J Pak Med Assoc. 2019;69(Suppl 3)(8):S4-S8.

[10] Smith CA, de Lacey S, Chapman M, et al. Effect of Acupuncture vs Sham Acupuncture on Live Births Among Women Undergoing In Vitro Fertilization: A Randomized Clinical Trial. JAMA. 2018;319(19):1990-1998. doi:10.1001/jama.2018.5336.

[11] Craig LB, Rubin LE, Peck JD, Anderson M, Marshall LA, Soules MR. Acupuncture performed before and after embryo transfer: a randomized controlled trial. J Reprod Med. 2014;59(5-6):313-320.

[12] Rashidi BH, Tehrani ES, Hamedani NA, Pirzadeh L. Effects of acupuncture on the outcome of in vitro fertilisation and intracytoplasmic sperm injection in women with polycystic ovarian syndrome. Acupunct Med. 2013;31(2):151-156. doi:10.1136/acupmed-2012-010198.

[13] Isoyama D, Cordts EB, de Souza van Niewegen AM, de Almeida Pereira de Carvalho W, Matsumura ST, Barbosa CP. Effect of acupuncture on symptoms of anxiety in women undergoing in vitro fertilisation: a prospective randomised controlled study. Acupunct Med. 2012;30(2):85-88. doi:10.1136/acupmed-2011-010064.

[14] Cui W, Li J, Sun W, Wen J. Effect of electroacupuncture on oocyte quality and pregnancy for patients with PCOS undergoing in vitro fertilization and embryo transfervitro fertilization and embryo transfer[J]. Zhongguo Zhen Jiu. 2011 Aug;31(8):687-91. Chinese. PMID: 21894688.

[15] Moy I, Milad MP, Barnes R, Confino E, Kazer RR, Zhang X. Randomized controlled trial: effects of acupuncture on pregnancy rates in women undergoing in vitro fertilization. Fertil Steril. 2011 Feb;95(2):583-7. doi: 10.1016/j.fertnstert.2010.05.024. Epub 2010 Jun 20. PMID: 20646688.

[16] Madaschi C, Braga DP, Figueira Rde C, Iaconelli A Jr, Borges E Jr. Effect of acupuncture on assisted reproduction treatment outcomes. Acupunct Med. 2010 Dec;28(4):180-4. doi: 10.1136/aim.2009.002022. Epub 2010 Oct 18. PMID: 20959311.

[17] Andersen D, Løssl K, Nyboe Andersen A, Fürbringer J, Bach H, Simonsen J, Larsen EC. Acupuncture on the day of embryo transfer: a randomized controlled trial of 635 patients. Reprod Biomed Online. 2010 Sep;21(3):366-72. doi: 10.1016/j.rbmo.2010.03.029. Epub 2010 Apr 7. PMID: 20638338.

[18] Li J, Cui W, Sun W. Effect of electroacupuncture treatment on pregnancy outcomes of IVF-ET in patients with kidney deficiency type PCOS [J]. Chinese Journal of Human Sexuality, 2009, 18(7): 28-30.

[19] Chen J, Liu LL, Cui W, Sun W. Effects of electroacupuncture on in vitro fertilization-embryo transfer (IVF-ET) of patients with poor ovarian response [J]. Zhongguo Zhen Jiu. 2009 Oct;29(10):775-9. Chinese. PMID: 19873910.

[20] So EW, Ng EH, Wong YY, Lau EY, Yeung WS, Ho PC. A randomized double blind comparison of real and placebo acupuncture in IVF treatment. Hum Reprod. 2009 Feb;24(2):341-8. doi: 10.1093/humrep/den380. Epub 2008 Oct 21. PMID: 18940896.

[21] Smith C, Coyle M, Norman RJ. Influence of acupuncture stimulation on pregnancy rates for women undergoing embryo transfer. Fertil Steril. 2006 May;85(5):1352-8. doi: 10.1016/j.fertnstert.2005.12.015. Epub 2006 Apr 5. PMID: 16600225.

[22] Westergaard LG, Mao Q, Krogslund M, Sandrini S, Lenz S, Grinsted J. Acupuncture on the day of embryo transfer significantly improves the reproductive outcome in infertile women: a prospective, randomized trial. Fertil Steril. 2006 May;85(5):1341-6. doi: 10.1016/j.fertnstert.2005.08.070. Epub 2006 Apr 5. PMID: 16600232.
